# Synthesis, Characterization and In Vitro Bioactivity of Magnetite Nanoparticles Obtained by Co-Precipitation

**DOI:** 10.3390/jfb17080393

**Published:** 2026-08-09

**Authors:** Marian Rascov, Angela Spoiala, Ludmila Motelica, Roxana-Doina Trusca, Cristina Chircov, Roxana Cristina Popescu, Otilia-Ruxandra Radacina, Vasile-Adrian Surdu, Denisa Ficai, Ovidiu-Cristian Oprea, Anton Ficai, Ecaterina Andronescu, Claudiu Stefan Turculet

**Affiliations:** 1 Department of Science and Engineering of Oxide Materials and Nanomaterials, Faculty of Chemical Engineering and Biotechnologies, National University of Science and Technology Politehnica Bucharest, Gh. Polizu 1–7, 011061 Bucharest, Romania; cristina.chircov@upb.ro (C.C.); anton.ficai@upb.ro (A.F.); 2 National Research Center for Micro and Nanomaterials, National University of Science and Technology Politehnica Bucharest, Splaiul Independentei 313, 060042 Bucharest, Romania; angela.spoiala@upb.ro (A.S.); ludmila.motelica@upb.ro (L.M.); roxana_doina.trusca@upb.ro (R.-D.T.); otilia.radacina@upb.ro (O.-R.R.); denisa.ficai@upb.ro (D.F.); ovidiu.oprea@upb.ro (O.-C.O.); 3 Research Center for Advanced Materials, Products and Processes, National University of Science and Technology Politehnica Bucharest, Splaiul Independentei 313, 060042 Bucharest, Romania; 4 Academy of Romanian Scientists, Ilfov St. 3, 050044 Bucharest, Romania; 5 Department of Life and Environmental Physics, National Institute for R&D in Physics and Nuclear Engineering “Horia Hulubei”, Reactorului St., 30, 077125 Magurele, Romania; 6 Department of Materials Science, Faculty of Materials Science and Engineering, Transilvania University of Brasov, 29 Eroilor Blvd., 500036 Brasov, Romania; vasile.surdu@unitbv.ro; 7 Department of Inorganic Chemistry, Physical Chemistry and Electrochemistry, Faculty of Chemical Engineering and Biotechnologies, National University of Science and Technology Politehnica Bucharest, Gh. Polizu 1–7, 011061 Bucharest, Romania; 8 Technical Sciences Academy of Romania, 26 Dacia Blvd., 010414 Bucharest, Romania; 9 Faculty of Medicine, “Carol Davila” University of Medicine and Pharmacy, Eroii Sanitari St. 8, 050474 Bucharest, Romania; claudiu.turculet@umfcd.ro; 10 General Surgery Department, Clinical Emergency Hospital of Bucharest, 105402 Bucharest, Romania

**Keywords:** Fe_3_O_4_ nanoparticles, co-precipitation synthesis, calcium–phosphate deposition, in vitro bioactivity, surface reactivity

## Abstract

Magnetite (Fe_3_O_4_) nanoparticles have been explored for biomedical applications. Their surface behavior in physiological-like environments has not been fully established. Fe_3_O_4_ nanoparticles obtained by chemical co-precipitation were exposed to simulated body fluid (SBF) for up to 28 days, and in vitro bioactivity was examined. Structural and surface changes were investigated using XRD, FTIR-ATR, SEM/EDS and thermal analysis, while pH and electrical conductivity measurements were used to monitor changes at the particle–solution interface. Magnetite was the main crystalline phase, and the main crystalline structure was preserved during SBF exposure within the detection limits. Progressive surface transformations occurred, including hydration/hydroxylation and formation of calcium–phosphate-containing surface deposits. Changes in pH and variations in electrical conductivity were interpreted as complementary indicators of interfacial processes. Cytotoxicity tests on L929 fibroblast cells showed that Fe_3_O_4__14SBF was well tolerated at 25–50 µg/mL, whereas higher concentrations reduced cell viability. These findings suggest that Fe_3_O_4_-based magnetic materials may be further considered for biomedical applications, provided that concentration-dependent cytocompatibility is considered.

## 1. Introduction

The term nanomaterials is commonly used for materials whose structure includes at least one dimension in the nanometer range, typically from 1 to 100 nm [1]. Over time, nanomaterials have been increasingly examined for different types of applications, with reported use in medicine [2,3,4], electronics [5,6,7], environmental research [8,9,10] and energy [11,12,13].

The increasing interest in nanomaterials for biomedical applications is mainly due to a high surface area-to-volume ratio [14], tunable surface chemistry [15], size- and shape-dependent physicochemical properties [1] and enhanced chemical reactivity [16,17].

Superparamagnetic iron oxide nanoparticles (SPIONs), a major subgroup of biomedical nanomaterials, are iron oxide nanoparticles that exhibit superparamagnetic behavior, with magnetic response limited to the applied external field and no remanence after field removal [18]. This behavior is useful for safe manipulation in vivo [19]. SPIONs have been explored for use as nanocarriers in targeted and triggered drug delivery, in hyperthermia therapy, in magnetic resonance imaging (MRI) as contrast agents, as well as for cell labeling and separation, tissue engineering and biosensing applications [20,21,22,23,24]. The drug delivery nanoplatforms are biocompatible and biodegradable and are ultimately processed and eliminated through physiological iron-metabolism pathways [25]. Due to their magnetic properties, the SPIONs can be guided to the targeted tumor site using an external magnetic field [24]. Despite their advantages, concerns regarding biocompatibility and potential toxicity still exist, which may induce serious risks to biological systems [22]. Applications in biosensing are continuously expanding.

Magnetite (Fe_3_O_4_) is one of the most investigated iron oxides for biomedical use [26]. The crystal structure is of inverse spinel type with cubic symmetry. Iron ions in both oxidation states are found in tetrahedral as well as octahedral coordination sites. This arrangement generates ferrimagnetic behavior at room temperature [15]. Magnetite shows relatively high saturation magnetization compared to other oxides [27]. Such properties enable magnetic guidance and concentration at selected sites [28]. The surface state and dispersion of magnetite nanoparticles play an important role in their biological compatibility, because aggregation may reduce the effective surface area and alter the interaction between particles and cells [29]. In many biomedical studies, surface coatings are used to limit aggregation, improve colloidal stability and support biocompatible behavior [30,31]. The behavior of uncoated or minimally processed magnetite nanoparticles remains relevant, because their surface is directly exposed to physiological-like media and may undergo surface reactions during immersion.

The performance of magnetite nanoparticles depends strongly on the synthesis route [15,32]. This is relevant because reaction conditions affect both the crystalline core and the surface characteristics that later influence interactions between particles and the surrounding medium. Co-precipitation remains the most widely used method. It allows for preparation in aqueous media under mild conditions [33]. Particle size is influenced by Fe^2+^/Fe^3+^ ratio and alkalinity [33]. The degree of crystallinity is progressively modified during the aging process and subsequent washing steps [15]. Nevertheless, co-precipitated magnetite often exhibits aggregation tendencies [34]. Particle aggregation reduces the effective surface area available for interactions with the surrounding medium [35,36]. Taken together, these aspects show that co-precipitated magnetite should be discussed not only in terms of synthesis route but also in terms of the surface and aggregation features generated by that route. These synthesis-dependent features provide the link between the preparation of magnetite nanoparticles and their subsequent evaluation in SBF. In particular, particle size, surface chemistry and aggregation state may influence how the particles hydrate, adsorb or exchange ions, undergo partial surface oxidation and contribute to the deposition of calcium and phosphate species at the particle–solution interface.

Simulated body fluid is widely employed as an in vitro model to screen the bioactivity of materials developed for biomedical applications, based on the ability of apatite to form on material surfaces during immersion [37]. SBF mimics the inorganic ionic composition of human blood plasma [38]. The ability of a material to induce apatite formation in SBF correlates with bone-bonding potential in vivo [39]. Kokubo’s simulated body fluid is currently recognized as an internationally accepted reference method for in vitro testing, being endorsed by the International Organization for Standardization for evaluating the apatite-forming capability of implant materials designed for direct contact with bone (ISO 23317:2014) [40,41].

The recent literature indicates that magnetite nanoparticles should not be regarded as completely inert during immersion in SBF [42]. After immersion in aqueous media, magnetite nanoparticles are expected to rapidly develop a hydrated interfacial layer at their surface [43]. This hydrated layer may represent an early stage in the interaction between Fe_3_O_4_ and the surrounding ionic medium. At the same time, uncoated Fe_3_O_4_ nanoparticles may undergo partial oxidation toward maghemite, while limited dissolution of iron species can occur during the first days of immersion [44,45]. These processes may further influence the nucleation or deposition of calcium–phosphate species. Amorphous calcium–phosphate layers have also been reported to form on magnetite surfaces, and their formation kinetics are influenced by pH and ionic strength [46,47].

The situation becomes more complex when magnetite is incorporated into bioactive matrices, because the observed SBF response reflects both the intrinsic surface chemistry of magnetite and the contribution of the surrounding matrix [42]. This distinction is important because, in composite systems, calcium–phosphate deposition may be influenced not only by magnetite but also by the chemistry and ion-release behavior of the matrix. For example, interfaces with silicate glasses can promote mineralization, whereas polymeric systems such as chitosan hydrogels may either favor or inhibit apatite nucleation, depending on composition, surface charge and ion mobility [48,49,50]. Many reported SBF responses of magnetite-containing systems are matrix-dependent and cannot be directly assigned to magnetite alone. Most available studies focus on functionalized magnetite or composite systems [51,52], while fewer reports address the intrinsic evolution of uncoated or minimally processed magnetite nanoparticles during SBF immersion [15,44,53]. In addition, data obtained after immersion periods longer than 14 days remain limited, although they are important for evaluating the longer-term stability and surface evolution of these particles [37]. Thus, there is a scientific gap that is not only related to the limited number of studies beyond 14 days but also to the limited number of time-resolved evaluations of minimally processed Fe_3_O_4_ nanoparticles performed outside composite or functionalized systems. The present study addresses this gap by evaluating minimally processed Fe_3_O_4_ nanoparticles during extended SBF immersion, with the aim of distinguishing the intrinsic structural, chemical and morphological response of magnetite from matrix-dependent effects reported for composite systems.

In this context, the present study investigates magnetite nanoparticles obtained by chemical co-precipitation and evaluates their in vitro bioactivity during immersion in SBF for 1, 3, 7, 14, and 28 days. Structural, morphological and surface-chemical changes were monitored using complementary techniques, including XRD, ED-XRF, FTIR-ATR, SEM-EDS, DLS and TG-DSC, while pH and conductivity measurements were used to follow interfacial processes occurring at the particle–solution interface. Cytotoxicity tests were performed to assess the biological tolerance of the selected SBF-treated sample. By focusing on minimally processed Fe_3_O_4_ nanoparticles rather than on composite or functionalized systems, this study provides a matrix-free reference for understanding the intrinsic behavior of magnetite in physiological-like media and supports the rational design of Fe_3_O_4_-based bioactive magnetic systems.

## 2. Materials and Methods

### 2.1. Materials

For synthesis only reagents of analytical quality were used. They were employed as supplied, without any additional treatment. Ferric chloride hexahydrate (FeCl_3_⋅6H_2_O) and ferrous chloride tetrahydrate (FeCl_2_⋅4H_2_O) were purchased from Sigma–Aldrich (St. Louis, MO, USA). Potassium hydroxide (KOH) was purchased from SC Silal Trading SRL (Bucharest, Romania). In the preparation of the aqueous solutions double-distilled water was used.

### 2.2. Method of Fe_3_O_4_ Synthesis

Magnetite nanoparticles were synthesized by the chemical co-precipitation method, which is considered one of the simplest and most efficient routes for obtaining iron oxide nanoparticles. This route is based on the controlled co-precipitation of ferric and ferrous salts, mixed in a stoichiometric ratio in aqueous solution. The reaction leads to the precipitation of magnetite, as described by Equation (1):

(1) $${Fe}^{2+}+{2Fe}^{3+}+{8OH}^{-}\to {Fe}_{3}O_{4}+{4H}_{2}O$$

Ferric chloride hexahydrate and ferrous chloride tetrahydrate were dissolved together in 100 mL of deionized water to obtain a mixed iron salt solution with a Fe^3+^/Fe^2+^ molar ratio of 2:1. The solution containing the iron salts was added slowly, dropwise, into an aqueous solution of KOH (0.3 mol/L) that was prepared separately.

During the mixing of the solutions a dark precipitate appeared. The precipitate was collected in a beaker, magnetically separated from the solution using a permanent magnet and washed several times with distilled water until the supernatant reached a neutral pH. The washed slurry was then dried at room temperature. This procedure broadly follows the method previously described by Eskandari and Hasanzadeh [54].

### 2.3. Characterization Methods

#### 2.3.1. X-Ray Diffraction

The structural characteristics of the samples were examined using a Shimadzu XRD-6000 diffractometer (Shimadzu, Kyoto, Japan) equipped with a Cu anode. The diffractograms were collected within the 2θ angle range between 20 and 80°. A scan rate of 2°/min and a step size of 0.02° were used. The X-ray diffraction patterns were indexed according to the reference data for magnetite, with a cubic inverse spinel structure and $Fd\bar{3}m$ space group, using the ICDD PDF-4+ database, reference code 04-025-3485.

Rietveld refinement was performed using whole-pattern fitting to estimate the crystallite size, lattice parameters and degree of crystallinity. A polynomial function was used to model the background, a pseudo-Voigt function to describe the diffraction peak profiles and the Caglioti function to model peak broadening. Crystallite size and microstrain were refined simultaneously during the whole-pattern Rietveld analysis to account for their respective contributions to diffraction peak broadening. The degree of crystallinity was calculated as the ratio between the integrated intensity assigned to the crystalline diffraction contributions and the total integrated intensity of the measured diffraction pattern.

#### 2.3.2. Energy Dispersive X-Ray Fluorescence

Elemental composition analysis of the synthesized magnetite, using a Shimadzu EDX-7000 spectrometer (Shimadzu Corporation, Kyoto, Japan), was performed at room temperature, under ambient atmosphere. For data acquisition the sample was placed inside of a Mylar-sealed holder that was irradiated using the Rh-anode X-ray tube, together with a standard collimator and automatic filter selection of the instrument. The spectrum was collected using the Al-U filter to restrict detection to the corresponding energy window. The semi-quantitative results are expressed as elemental mass percentages to more accurately reflect the composition of the obtained magnetite.

#### 2.3.3. Fourier-Transform Infrared Spectroscopy

The structural characterization of the magnetite samples was studied using an Invenio-R spectrometer (Bruker Optik GmbH, Ettlingen, Germany) equipped with an attenuated total reflection accessory and a DLaTGS detector (Bruker Optik GmbH, Ettlingen, Germany). Spectra were collected in the 4000–400 cm^−1^ range at a 4 cm^−1^ resolution, with an accuracy of 0.1 cm^−1^, a photometric precision of 0.1% T, and 128 scans. Spectral processing was performed using the OPUS software, version 8.8 (Bruker Optik GmbH, Ettlingen, Germany) applying atmospheric corrections, normalizations and baseline adjustments.

#### 2.3.4. Scanning Electron Microscopy—Energy Dispersive X-Ray Spectroscopy

The morphology of the samples was examined using an Inspect F50 scanning electron microscope (Thermo Fisher Scientific, Eindhoven, The Netherlands). SEM images were used for qualitative evaluation of aggregate morphology and surface changes after SBF immersion, rather than for statistical particle size distribution analysis. Small quantities of powder were mounted on carbon-coated adhesive tabs and introduced into the microscope chamber. The images were recorded at different magnifications using a 30 kV electron beam and secondary electron detection. The coupled EDS detector attached to the same instrument allowed for the simultaneous collection of elemental information during imaging.

#### 2.3.5. Dynamic Light Scattering

For DLS analysis, the powders were dispersed in deionized water (1 mg/mL, pH close to 7). To break up large agglomerates and ensure a uniform dispersion, the solutions were sonicated for 10 min. The samples were transferred with a pipette into the cell of the DelsaMax Pro instrument (Beckman Coulter, Brea, CA, USA), taking care to avoid air bubbles. Each dispersion was analyzed in triplicate.

#### 2.3.6. Thermogravimetric Analysis

Thermal analysis (TG-DSC) of the magnetite samples was performed using a STA 449C Jupiter apparatus from Netzsch (NETZSCH-Gerätebau GmbH, Selb, Germany). The samples were placed in an open crucible made of alumina and heated with 10 K/min from room temperature up to 900 °C, under the flow of 50 mL/min dried air. An empty alumina crucible was used as reference.

#### 2.3.7. In Vitro Bioactivity Evaluation

The bioactivity of the magnetite powder was evaluated by in vitro immersion in simulated body fluid (SBF). The SBF used for the immersion tests was prepared after Kokubo’s method [55]. For each sample, a quantity of 0.5 g was immersed in 150 mL of SBF in closed polypropylene containers and maintained at 36 ± 1 °C for periods ranging from 1 up to 28 days.

Electrical conductivity and pH changes were studied periodically throughout immersion. At the end of the immersion time, the samples were examined for evaluating the formation of apatite.

#### 2.3.8. Cell Viability Assay

L929 fibroblast cells (NCTC clone 929 [L cell, L-929, derivative of Strain L], ATCC CCL-1^TM^) were cultured in Dulbecco’s Modified Eagle Medium (DMEM) containing 10% fetal bovine serum and 1% Penicillin–Streptomycin at 37 °C, 5% CO_2_ and 90% humidity. The cells were seeded in 96-well plates at 5000 cells per 100 µL and were left to attach for 24 h.

Cell viability testing was performed on the Fe_3_O_4__14SBF sample, corresponding to magnetite nanoparticles immersed in SBF for 14 days. The Fe_3_O_4__14SBF sample was considered representative of surface modifications occurring during the bioactivity process. Fe_3_O_4__14SBF was selected because it represents an intermediate stage of the bioactivity process, with evident surface modification and calcium–phosphate deposition, but before the more advanced surface coverage observed after 28 days. The assay was designed to evaluate the biological response to a representative surface-modified magnetite sample, rather than to compare the cytocompatibility of all SBF immersion times. After incubation, the culture medium was replaced with a fresh medium containing Fe_3_O_4__14SBF at concentrations between 200 and 25 µg/mL (two-fold dilutions). After 5 days of incubation cell viability was studied.

Cell viability was assessed using the MTT assay. Following exposure, the medium was removed and replaced with MTT solution (10% MTT stock, 5 mg/mL in PBS, diluted in complete DMEM), and cells were incubated for 2 h. Formazan crystals were dissolved in DMSO, and absorbance was measured at 570 nm. Viability was expressed relative to untreated negative controls (100%). For the interpretation of the results, measurements above the 70% level were treated as non-cytotoxic. The positive control was a 1% Triton X-100 solution.

## 3. Results and Discussion

### 3.1. X-Ray Diffraction

**Figure 1:**
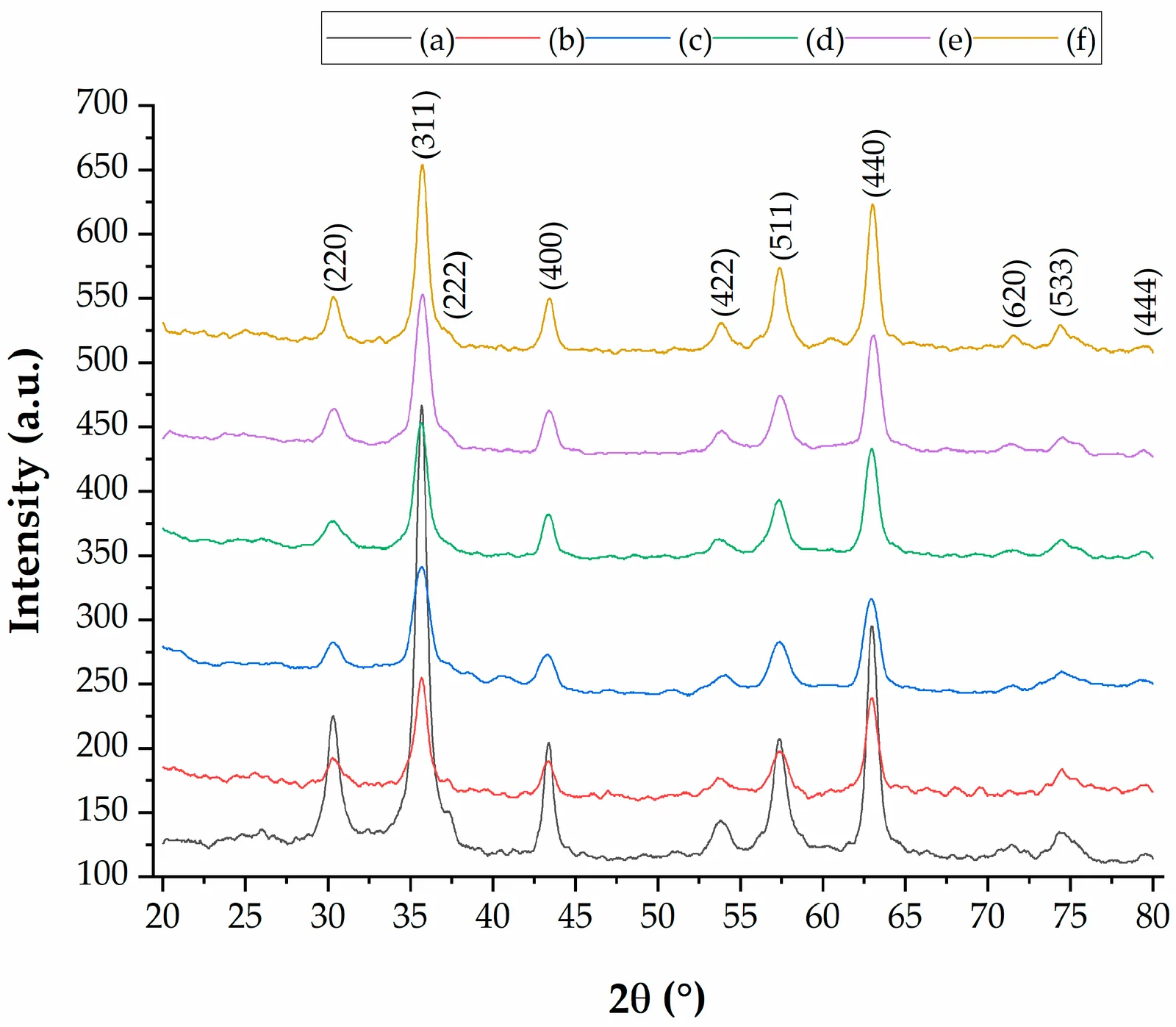
XRD diffractograms acquired for Fe_3_O_4_ powder before immersion in SBF (a) and after immersion in SBF for 1 day (b), 3 days (c), 7 days (d), 14 days (e) and 28 days (f). The diffractograms are vertically offset for clarity.

The crystalline structure of pristine magnetite and of the samples immersed in simulated body fluid (SBF) for different time intervals was investigated by X-ray diffraction (Figure 1).

In all XRD patterns, reflections consistent with a cubic spinel iron oxide structure were identified and indexed to Fe_3_O_4_ with the $Fd\bar{3}m$ space group, according to the ICDD PDF-4+ database, reference code 04-025-3485. The main peaks correspond to the (220), (311), (222), (400), (422), (511), (440), (620), (533) and (444) crystallographic planes. The XRD patterns were indexed to magnetite, and no additional crystalline iron oxide phases were detected. Because magnetite and maghemite have closely related spinel structures with overlapping diffraction peaks, XRD alone cannot completely exclude minor surface-oxidized or poorly crystalline iron oxide/oxyhydroxide species. Magnetite is considered the main crystalline phase of the synthesized powder, within the detection limits of XRD.

During SBF immersion, no new diffraction peaks were observed, indicating that the main crystalline spinel structure was preserved throughout the investigated period within the detection limits of XRD.

The structural parameters obtained by whole-pattern Rietveld refinement are summarized in Table 1.

The degree of crystallinity decreased from 57.98% for pristine Fe_3_O_4_ to 46.82% after 3 days of SBF immersion and subsequently increased to 57.96% after 28 days. The initial decrease may reflect a greater contribution from poorly crystalline or amorphous surface species formed during the early stages of immersion. Together with the FTIR-ATR, SEM-EDS and ED-XRF findings, this trend is consistent with the formation of phosphate-containing surface deposits that were insufficiently crystalline or abundant to generate distinct diffraction peaks. The diffraction data alone cannot establish the chemical identity of the amorphous contribution. The subsequent recovery of crystallinity may indicate that the contribution of coherently diffracting spinel domains became more pronounced at longer immersion times, although the underlying mechanism cannot be resolved from the present XRD data alone.

The average crystallite size showed a similar non-monotonic variation, decreasing from 9.94 ± 1.83 nm for pristine Fe_3_O_4_ to 6.40 ± 1.00 nm after 3 days and increasing to 11.18 ± 3.01 nm after 28 days. These values represent coherently diffracting domains rather than the dimensions of the aggregated particles observed by SEM and should therefore not be interpreted as direct evidence of particle growth or dissolution. Although crystallite size and microstrain were refined simultaneously, the transient decrease in apparent crystallite size after 3 days and the subsequent increase may reflect changes in the relative contributions of crystalline and poorly crystalline domains rather than dissolution and recrystallization. This interpretation should be regarded as a possible explanation rather than a definitive mechanism. The refined lattice parameter varied only slightly, between 8.344 and 8.356 Å, with no monotonic dependence on immersion time. Minor differences in the lattice parameter may reflect fitting uncertainty, surface oxidation, structural defects or microstrain. The present data do not support a systematic lattice expansion or a specific interfacial strain mechanism. Together with the absence of additional crystalline diffraction peaks, these results indicate that the cubic spinel structure remained largely preserved during SBF immersion.

### 3.2. Energy Dispersive X-Ray Fluorescence

The elemental composition of the samples was investigated by ED-XRF. A semi-quantitative analysis was performed for pristine magnetite and magnetite immersed in SBF for 1, 14 and 28 days. The selected elemental contents are summarized in Table 2. The corresponding ED-XRF spectra are provided in Figure S1 in the Supplementary Materials.

Fe remained the major detected element in all samples, decreasing from 99.527 wt.% in pristine Fe_3_O_4_ to 96.210 wt.% after 28 days of SBF immersion. This decrease was accompanied by progressive increases in P and Ca, which reached 2.209 wt.% and 1.152 wt.%, respectively, after 28 days. This compositional evolution is consistent with the progressive development of Ca–P-containing surface deposits during SBF immersion.

The P/Ca mass ratio decreased from 2.48 after 1 day to 2.25 after 14 days and 1.92 after 28 days, indicating progressive Ca enrichment of the deposited material. The initially higher P/Ca mass ratio may indicate phosphate-rich surface modification during the early stage of SBF immersion. This behavior may reflect the preferential interaction of phosphate species with the hydrated or hydroxylated magnetite surface, possibly through Fe–O–P surface complexes or iron–phosphate-like species. As the immersion time increased, the Ca content also increased, while the P/Ca mass ratio decreased, indicating progressive Ca enrichment of the surface deposits. This trend is consistent with the gradual development of Ca–P-containing deposits following the initially P-rich surface modification. At longer immersion times, these deposits may evolve toward more Ca-rich calcium–phosphate species. Because ED-XRF provides elemental information only, the formation of a specific calcium–phosphate phase, including apatite or hydroxyapatite, cannot be confirmed from these results alone. The ED-XRF findings should be interpreted together with the complementary characterization results.

The higher relative standard deviation observed for phosphorus is most likely related to its low concentration and weaker ED-XRF signal compared with the more abundant detected elements. Since phosphorus is associated with Ca–P species formed during SBF immersion, local variations in the amount of deposited material may also contribute to the observed variability. The phosphorus values were interpreted semi-quantitatively.

**Figure 2:**
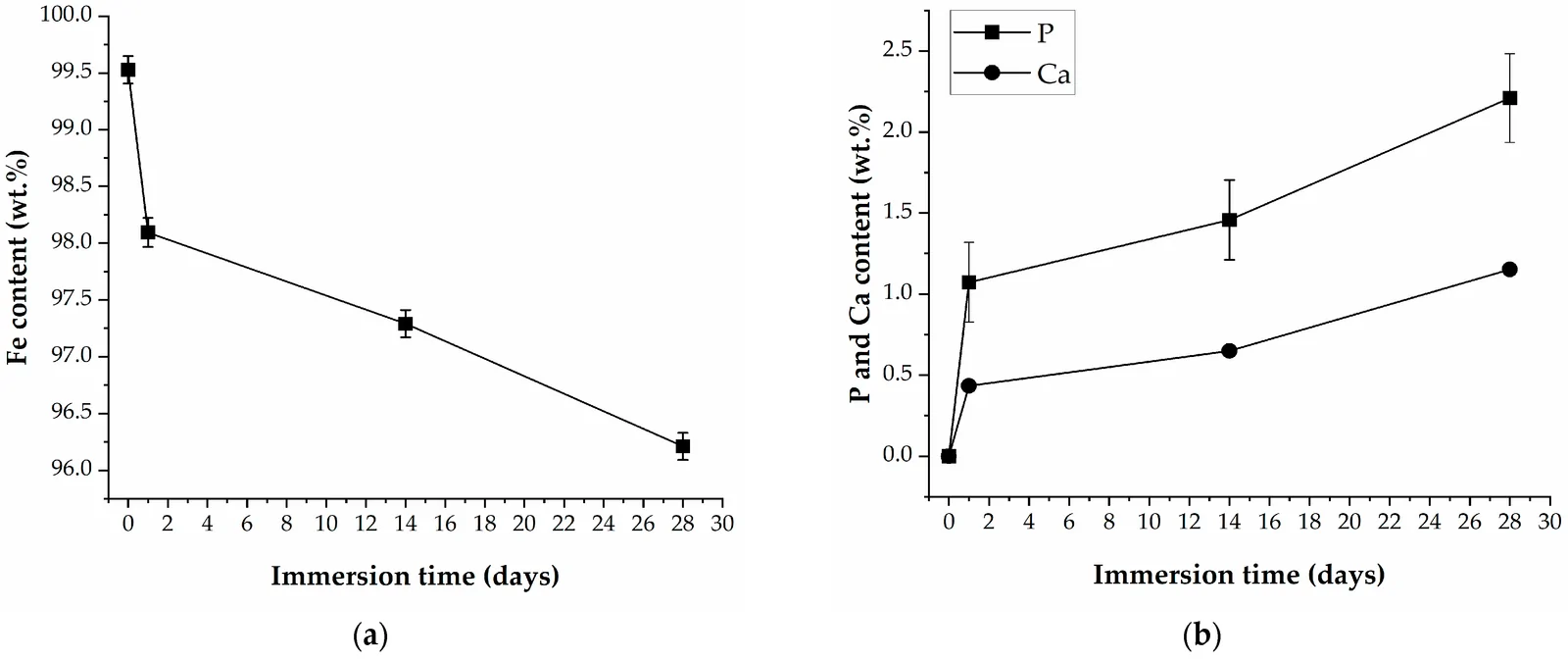
Evolution of Fe, P and Ca content as a function of SBF immersion time: (**a**) Fe content and (**b**) P and Ca content. The value at 0 days corresponds to pristine Fe_3_O_4_. Data are presented as means ± SD. Where not visible, error bars are smaller than the symbols.

A graphical evolution of Fe, P and Ca contents is shown in Figure 2.

The ED-XRF results show a systematic decrease in the relative Fe content and progressive increases in P and Ca during SBF immersion, consistent with the development of Ca–P-containing surface deposits.

### 3.3. Fourier-Transform Infrared Spectroscopy

FTIR analysis was used to monitor the surface changes of magnetite samples. For all samples, the usual bands in the 400–650 cm^−1^ region were identified and assigned to Fe-O stretching vibrations of the iron oxide structure. Fe–O bands are correlated with two kinds of positions that Fe can occupy in the crystal structure: tetrahedral and octahedral sites. The bands from 435 (octahedral sites of magnetite), 535 (tetrahedral sites of magnetite), 605, 615 and 623 cm^−1^ are characteristic for Fe–O stretching and remain present in all spectra, supporting the preservation of the Fe–O framework during SBF immersion [56,57,58,59,60,61,62,63,64]. FTIR-ATR alone cannot definitively discriminate between magnetite, maghemite and minor surface-oxidized or hydroxylated iron oxide species. These bands were considered supporting evidence for the iron oxide structure, rather than as proof of phase-pure magnetite.

**Figure 3:**
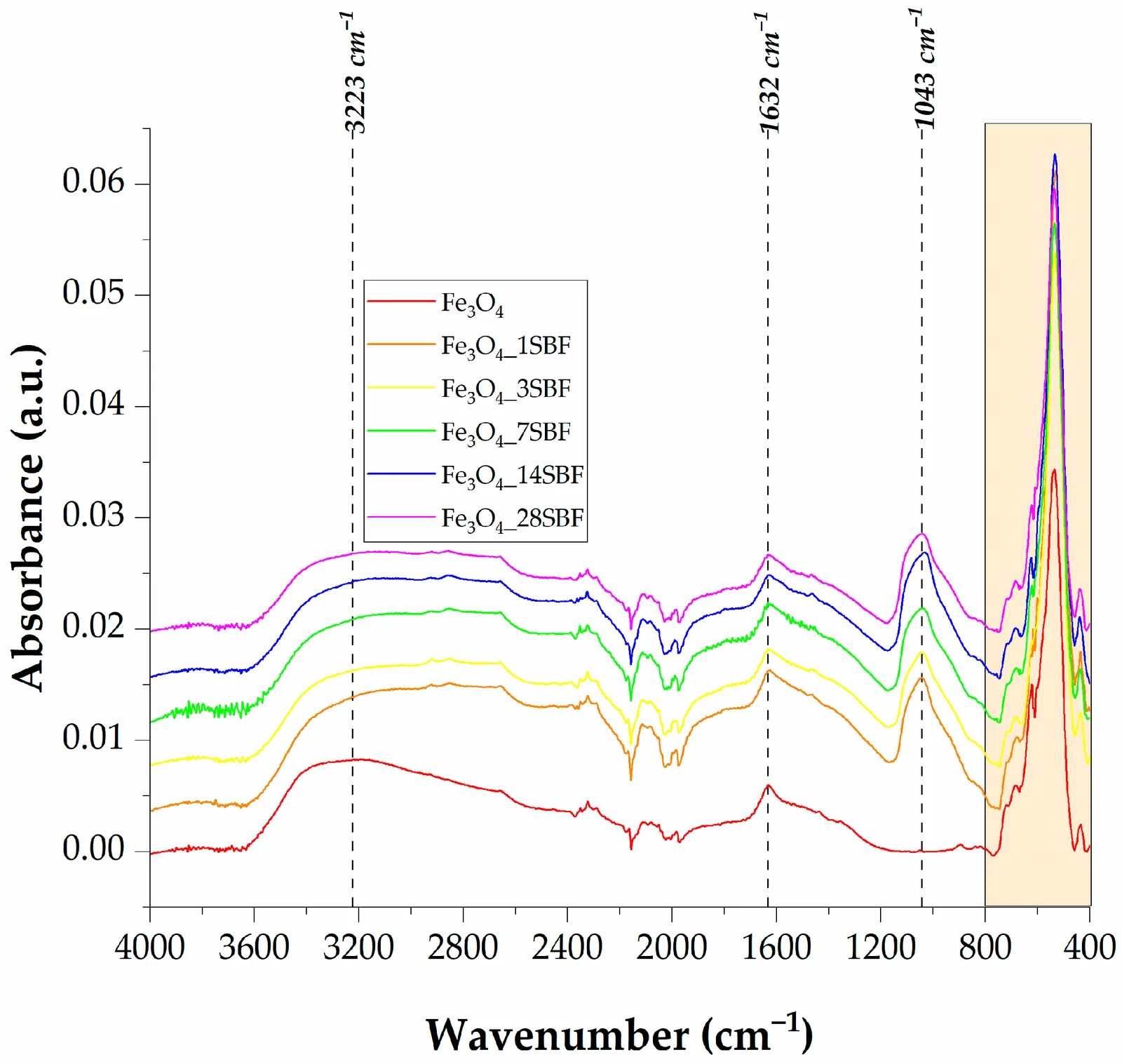
FTIR-ATR spectra of pristine Fe_3_O_4_ nanoparticles and Fe_3_O_4_ samples after immersion in SBF for 1, 3, 7, 14, and 28 days, recorded in the 4000–400 cm^−1^ wavenumber range. The spectra are vertically offset for clarity. The orange-shaded region indicates the spectral range enlarged in Figure 4.

**Figure 4:**
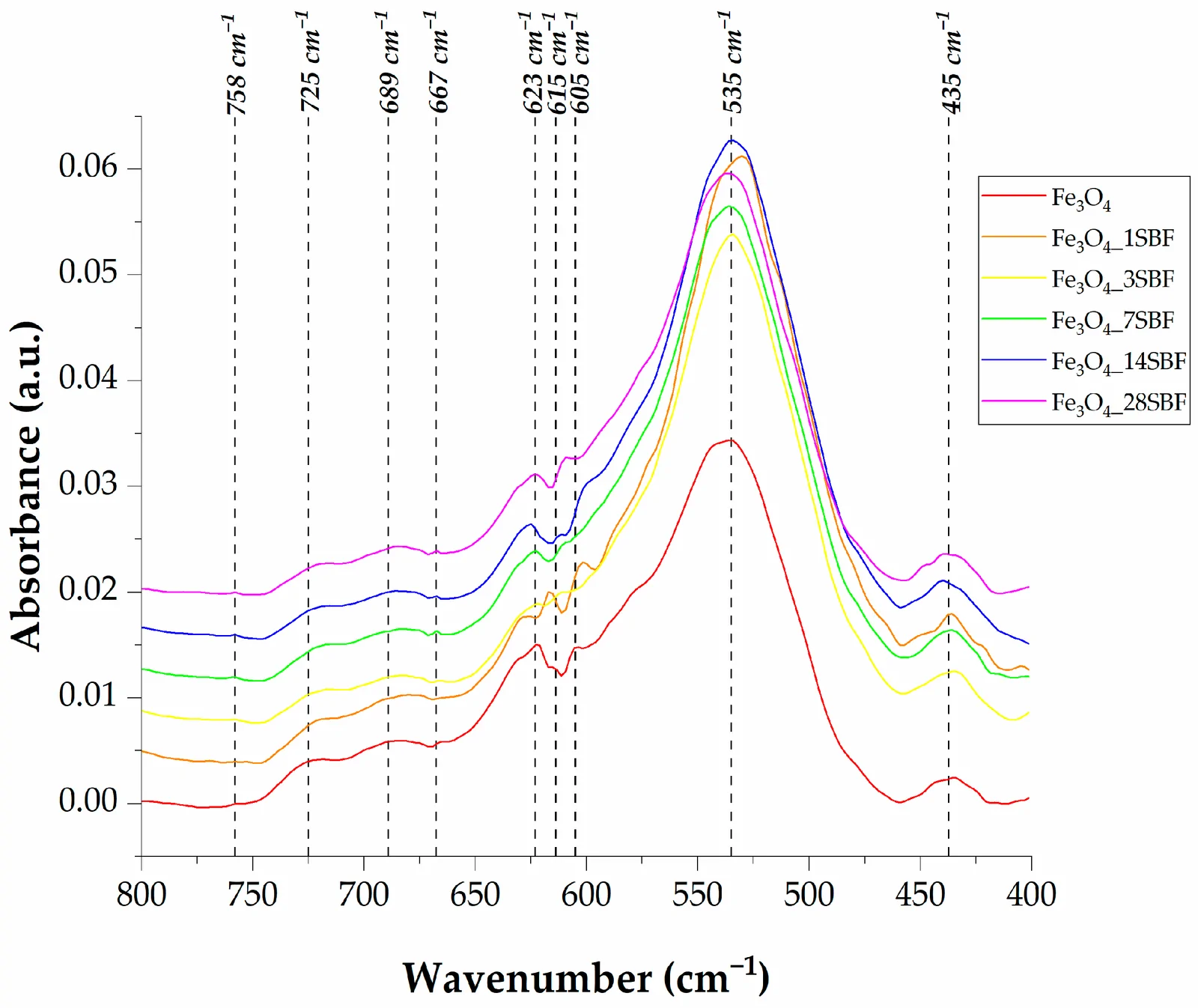
Magnified FTIR-ATR spectra of pristine Fe_3_O_4_ nanoparticles and Fe_3_O_4_ samples after immersion in SBF for 1, 3, 7, 14, and 28 days, showing the 800–400 cm^−1^ wavenumber region. The spectra are vertically offset for clarity.

Regarding the 650–800 cm^−1^ region, weak bands were identified at 667, 689, 725 and 758 cm^−1^, visible in all recorded spectra, including pristine magnetite. Since these bands were already present before SBF immersion and do not show a systematic increase with immersion time, they cannot be directly associated with SBF-induced surface mineralization. The band at 667 cm^−1^ is assigned to a possible contribution from the ν_2_ bending vibration of atmospheric/background CO_2_, commonly reported around 667–668 cm^−1^ in FTIR spectra [65,66]. The weak band at 689 cm^−1^ is tentatively related to a surface-oxidized iron oxide contribution, as a similar weak ATR-FTIR band at 687 cm^−1^ has been associated with the oxidation of magnetite [64]. The band at 725 cm^−1^ is tentatively assigned to weak metal–oxide-related stretching/bending vibrations, since a similar FTIR band at 721 cm^−1^ has been reported for iron oxide nanoparticles [67]. The band at 758 cm^−1^ is interpreted as part of the same weak, overlapping surface-related vibrational region, most likely associated with low-intensity Fe–O/Fe–O–Fe vibrations from an oxidized or hydroxylated iron oxide surface layer. The bands at 689, 725 and 758 cm^−1^ are considered weak surface-related features of the pristine magnetite powder, without being specific markers of a newly formed phase. Due to their low intensity and overlapping character, FTIR-ATR alone does not allow for an unambiguous assignment of these features to a specific iron oxide phase or quantification of possible magnetite or surface-oxidized contributions [68]. Accordingly, these bands were not used as evidence of SBF-induced mineralization. The corresponding full-range and magnified FTIR-ATR spectra are presented in Figure 3 and Figure 4, respectively.

The most relevant and interesting changes appear in the phosphate region, between 900 and 1200 cm^−1^. At 1043 cm^−1^ a peak is observed for the samples immersed in SBF and is associated with the asymmetric ν_3_ stretching of P–O in phosphate groups, suggesting the formation of phosphate-related surface species on the sample surface [69,70,71]. Since calcium–phosphate/apatite formation during SBF immersion is widely used as an indicator of in vitro bioactive behavior, the appearance of this phosphate-related band supports the surface reactivity of the sample in SBF [37,40,53,55].

The FTIR-ATR evidence for calcium–phosphate/apatite formation should be interpreted with caution. Additional phosphate-related bands typically reported for calcium–phosphate or apatite phases, such as the ν_1_(PO_4_^3−^) vibration near 960–968 cm^−1^ and the ν_4_(PO_4_^3−^) bending modes in the 560–605 cm^−1^ region, are not clearly resolved in the present spectra [69]. Their absence or poor definition may be related to the low amount, surface-confined distribution and/or poor crystallinity of the Ca–P deposits formed during SBF immersion [37]. Moreover, the 560–605 cm^−1^ region overlaps with the intense Fe–O vibrations of magnetite, which limits the unambiguous identification of phosphate bending modes by FTIR-ATR alone [64,68]. The band at 1043 cm^−1^ is considered supportive evidence of phosphate-related surface species but not standalone proof of a well-crystallized apatite phase. The mineralization process is therefore discussed based on complementary analyses.

Another important band appears at 1632 cm^−1^. This is associated with the bending vibration of H-O-H from adsorbed water [72,73,74]. It is present in all samples, indicating the persistence of adsorbed water or surface hydration during the investigated period.

Between 3000 and 3600 cm^−1^, in the top region of the spectrum, a wide absorption band appears at around 3223 cm^−1^, corresponding to ν(O–H) stretching of surface hydroxyl groups and physically adsorbed water [75,76,77]. This assignment is consistent with the hydrated and hydroxylated surface of magnetite nanoparticles in aqueous media, where surface Fe–OH groups participate in interfacial reactions [78,79].

The FTIR-ATR spectra show that the Fe–O bands preserve their characteristic positions, supporting the structural stability of the Fe_3_O_4_-based samples during SBF immersion. At the same time, the appearance of phosphate-related features indicates surface interaction with SBF and supports the formation of phosphate-related surface species. The FTIR-ATR results should be considered supporting evidence for the preservation of the Fe–O framework and the formation of phosphate-related surface species, rather than definitive proof of phase-pure magnetite or a well-crystallized apatite layer.

### 3.4. Scanning Electron Microscopy—Energy Dispersive X-Ray Spectroscopy

**Figure 5:**
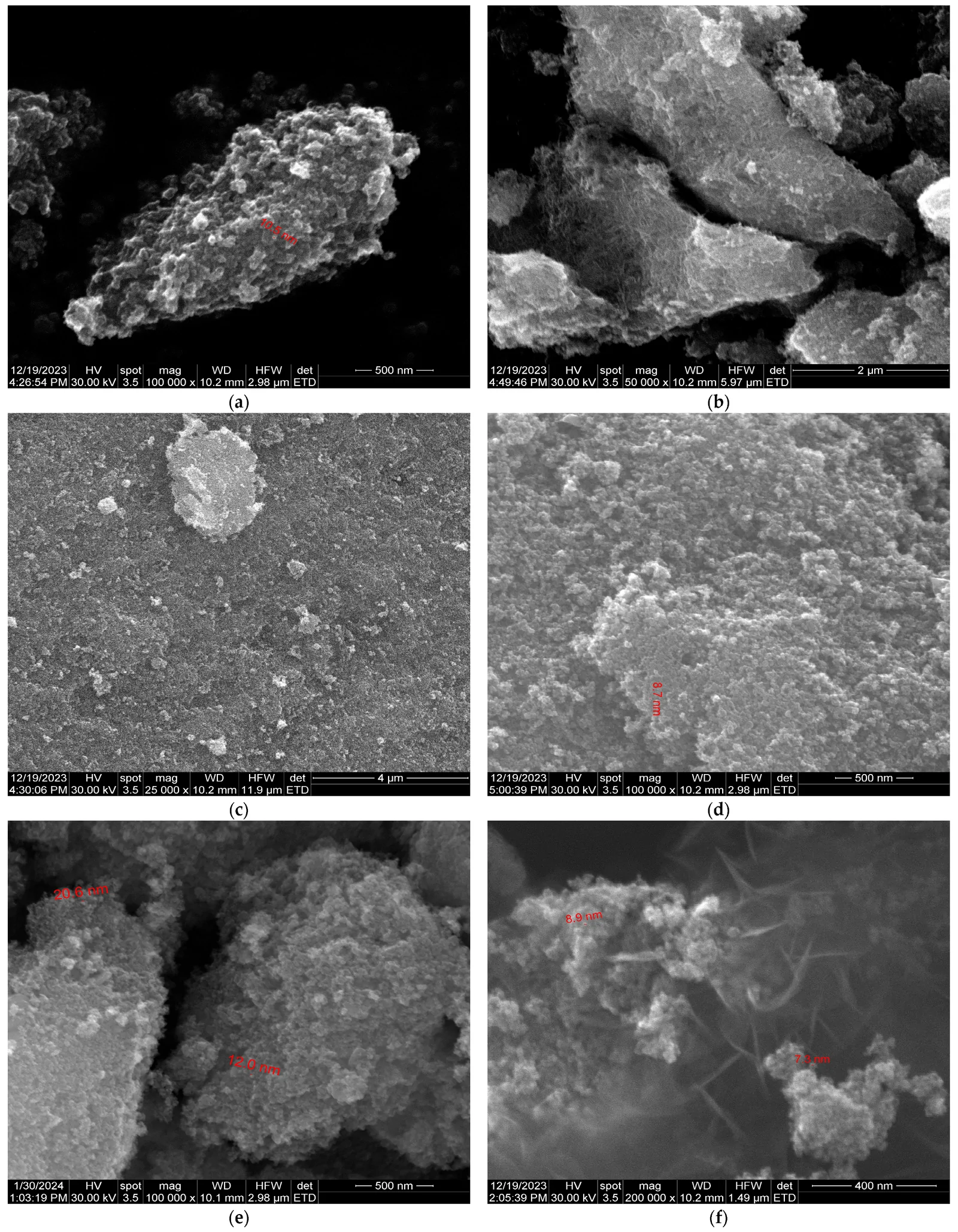
SEM images of the Fe_3_O_4_ powder: before (**a**) and after immersion in SBF for 1 day (**b**), 3 days (**c**), 7 days (**d**), 14 days (**e**) and 28 days (**f**). The red annotations indicate local illustrative measurements of selected surface features and should not be interpreted as representative particle sizes.

In Figure 5 the SEM images of magnetite particles before and after immersion in SBF for 1 to 28 days are shown, recorded at different magnifications.

Based on the SEM images, the obtained magnetite powder appears to be composed of tiny and irregular particles that join into aggregates. Even at higher magnifications, the aggregates remain visible and can be distinguished. Because of the strong aggregation and the limited number of clearly distinguishable individual particles, SEM images were used only for qualitative morphological evaluation and not for statistical particle size distribution analysis. The pristine magnetite appears as irregular aggregates, having a compact structure and relatively smooth surfaces. No secondary surface deposits are visible at this stage.

After a single day of immersion in SBF medium, the magnetite particles preserve the initial morphology. Fine granular formations begin to appear on the aggregate surfaces in a discontinuous manner. This may indicate early surface modification after SBF immersion. For the samples soaked in SBF for 3 days a morphological change is observed. The fine granular formations tend to become more frequent and to form a thin and discontinuous layer, spreading over larger surface areas. The presence of fine granular features is consistent with the early development of surface deposits during SBF immersion. The changes become more pronounced after 7 days. As the immersion time increases, small clusters become more visible on the magnetite surface. For this reason, the aggregates have a rougher appearance at the contact zones.

After 14 days, the surface appears more covered and rougher, suggesting a more advanced stage of surface deposition. After 28 days, the aggregates show a more covered and roughened surface. These morphological changes suggest a more pronounced surface deposition after prolonged SBF immersion. The SEM images alone cannot unambiguously prove the formation of a mature apatite layer. The SEM observations were considered as qualitative morphological support and were interpreted together with the complementary characterization results.

**Figure 6:**
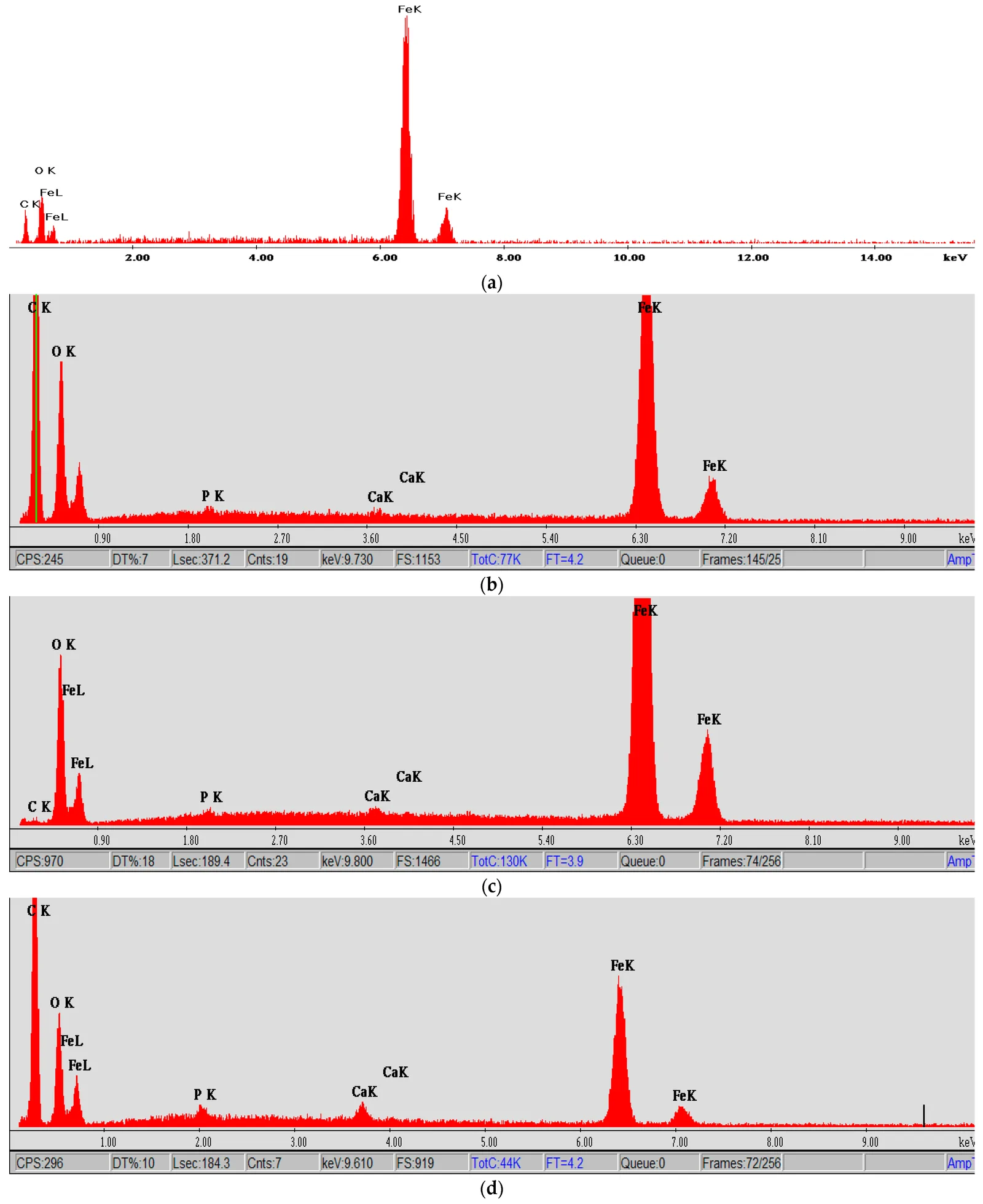

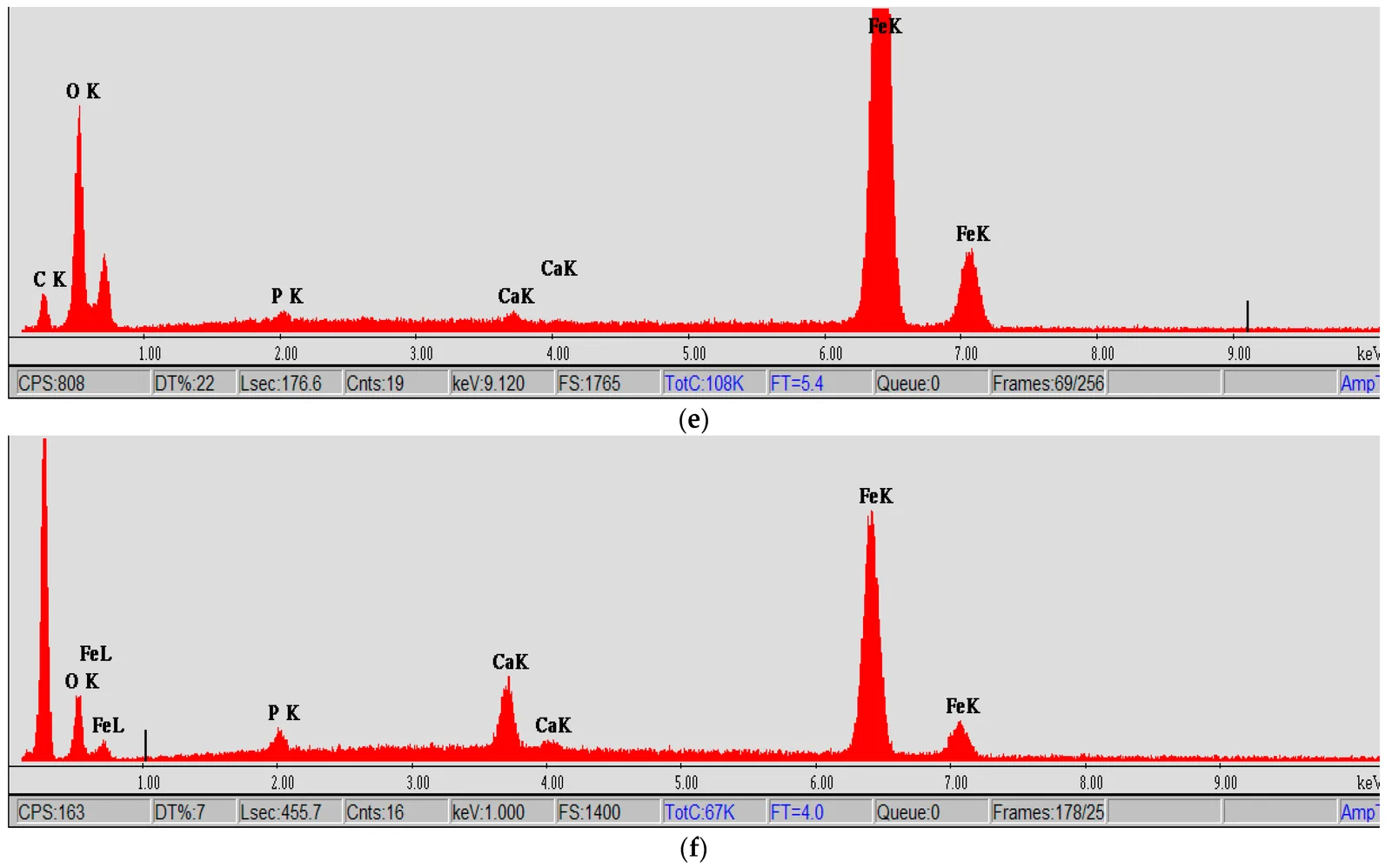
Representative EDS spectra of the Fe_3_O_4_ powder: before (**a**) and after immersion in SBF for 1 day (**b**), 3 days (**c**), 7 days (**d**), 14 days (**e**) and 28 days (**f**). The spectra are intended for qualitative elemental identification and not for direct quantitative comparison between samples. The red traces represent the EDS spectra, while the blue text and green markers are software-generated display elements and do not represent additional analytical data.

The surface composition of the samples was qualitatively evaluated using EDS spectra for the pristine magnetite and for the samples immersed in SBF for 1–28 days. The corresponding representative spectra are shown in Figure 6. Because the spectra are presented using different intensity scales and were not normalized for quantitative comparison, they were used only for qualitative elemental identification and not for direct comparison of signal intensities between samples. Based on the EDS spectra, iron and oxygen were the main elements identified for pristine magnetite. Calcium, phosphorus and other elements were not observed in the pristine powder, indicating the absence of detectable Ca-P surface deposits prior to SBF immersion.

The spectrum corresponding to the first day of immersion reveals weak calcium and phosphorus signals. These signals suggest an early interaction between the magnetite surface and ions from the SBF medium. For the sample immersed in SBF for 3 days, weak Ca and P signals are still observed. These results suggest the presence of Ca-P-containing species on the sample surface, although the spectra do not allow for a quantitative evaluation of layer growth. After 7 days of immersion in SBF, Ca and P signals remain visible, supporting the presence of Ca-P-containing surface deposits. After 14 and 28 days, the EDS spectra continue to show Ca and P signals, consistent with the presence of Ca–P-containing deposits on the magnetite surface after prolonged SBF immersion. The EDS spectra support the presence of Ca and P after SBF immersion, while the progression of Ca–P deposition was interpreted together with ED-XRF, FTIR-ATR and SEM observations.

### 3.5. Dynamic Light Scattering

Regarding DLS analysis, the pristine magnetite has a mean hydrodynamic diameter of 216.03 ± 6.06 nm and a PDI equal to 0.22 ± 0.02. These values indicate that the particles are dispersed in water mainly as moderately uniform aggregates rather than as isolated primary nanoparticles. The much larger hydrodynamic size compared with the primary particle size is common for uncoated Fe_3_O_4_ in aqueous media, where particle clusters and their surrounding hydration layers contribute to the measured diameter [80,81].

A zeta potential of +19.95 ± 3.56 mV indicates moderate colloidal stability, suggesting that the dispersion is not highly stable but retains sufficient surface charge to reduce rapid uncontrolled agglomeration. This positive surface charge is consistent with the amphoteric behavior of magnetite, where Fe–OH groups at the surface may become protonated under aqueous conditions [78,79].

The DLS results indicate that pristine magnetite forms hydrated, moderately aggregated and positively charged particles in water. These features are relevant for SBF immersion because particle aggregation, hydration and surface charge can influence the accessibility of the particle surface and its interaction with ions from the surrounding medium. The results of the DLS analysis are presented in Table 3.

### 3.6. Thermogravimetric Analysis

**Figure 7:**
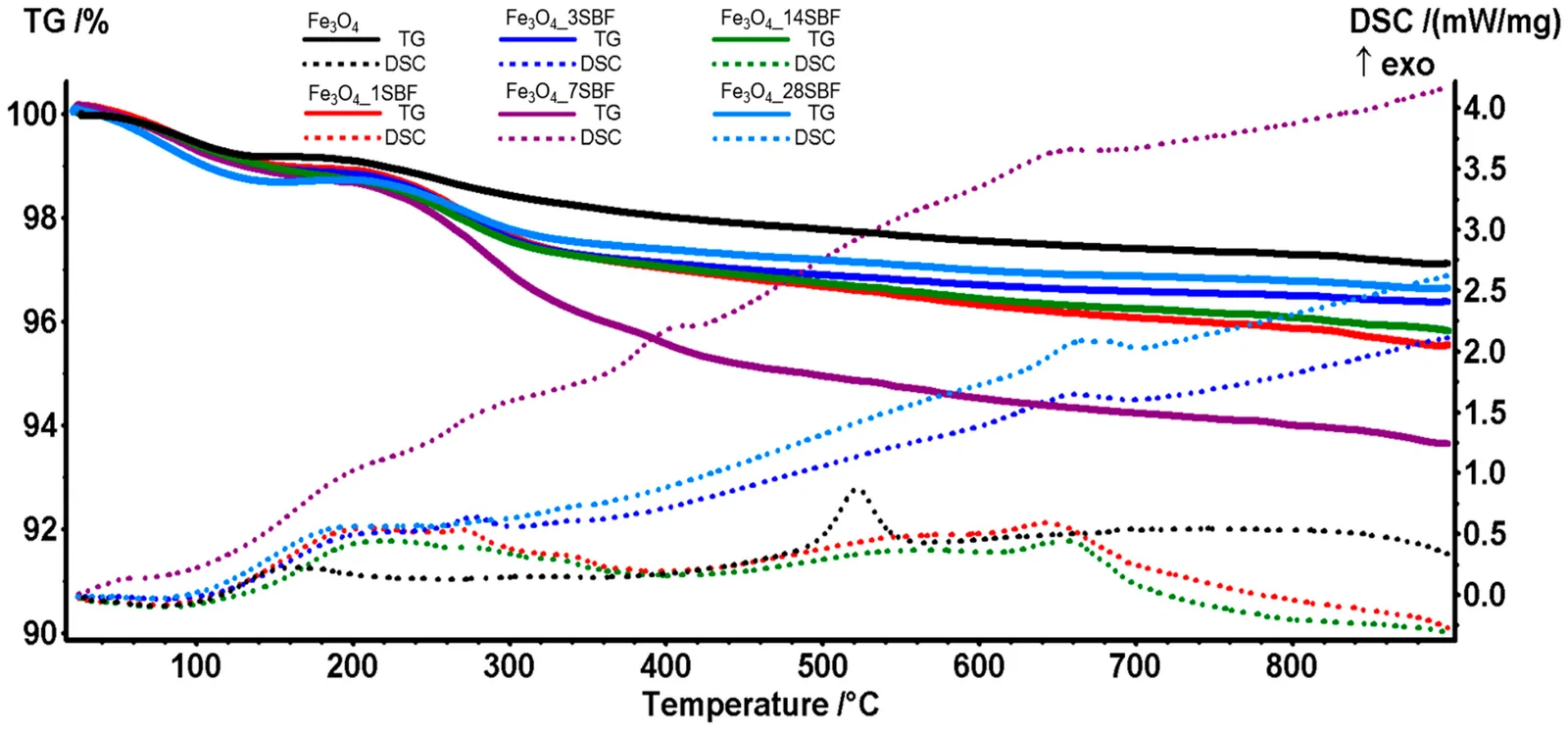
TG/DSC curves of the magnetite-based samples in the 20–900 °C range.

Figure 7 shows the TG/DSC curves of the magnetite samples measured between room temperature and 900 °C. A magnified view of the low-temperature region is provided in Figure S2 in the Supplementary Materials.

Up to about 180 °C, all samples show a small mass loss caused by the removal of residual water molecules physically adsorbed on the particle surface [82]. The recorded values range from 0.85% to 1.31% for the pristine magnetite and for the SBF-treated samples. The process is accompanied by a weak endothermic event on the DSC curve, with a minimum between 62 and 80 °C.

Above 180 °C, the pristine Fe_3_O_4_ sample exhibits a slow and continuous mass decrease. This is attributed to the condensation of terminal –OH groups and the release of additional water (1.34% up to 480 °C and another 0.69% up to 900 °C) [83]. Below 400 °C, the DSC curve shows several weak overlapping endothermic and exothermic effects. These overlapping thermal events may be associated with the elimination of surface hydroxyl groups and with oxidation processes involving Fe^2+^/Fe^3+^ species during heating in air [84,85].

A strong exothermic peak at 522.0 °C can be associated with iron oxide oxidation/phase transformation processes, including the commonly reported transition from maghemite to hematite (γ-Fe_2_O_3_ to α-Fe_2_O_3_) [86]. When magnetite is protected by an inert or encapsulating shell, this transition may be suppressed, and the DSC peak may disappear, as previously reported [87,88]. The residual mass of the pristine Fe_3_O_4_ sample is 97.12%, and the remaining solid is consistent with an iron oxide residue formed after heating in air [89]. This residual mass value was used to estimate the surface load (%) for all Fe_3_O_4__xSBF samples, as listed in Table 4. The principal numeric data from the thermal analysis are presented in Table 4.

Compared with the control sample, the Fe_3_O_4__xSBF materials show two notable differences. The first appears above 180 °C, where a higher mass loss is recorded for all SBF-treated samples. This effect may be associated with the partial oxidation/decomposition of surface species adsorbed during immersion in SBF. The DSC curves in this region also show additional exothermic contributions absent in the pristine sample.

The second modification occurs above 480 °C. For the Fe_3_O_4__xSBF samples, the exothermic event associated with iron oxide oxidation/phase transformation shifts to higher temperatures (644.9–669.1 °C). This shift may be related to the removal or transformation of surface species introduced during SBF immersion between 500 and 600 °C, as indicated by the broad and asymmetric exothermic signal observed in this interval. Similar shifts of the exothermic peak associated with the iron oxide oxidation/phase transformation have been reported previously in the literature [90]. All these features are consistent with the thermal behavior of Fe_3_O_4_-based samples, but they do not provide definitive phase discrimination between magnetite, maghemite and minor surface-oxidized or hydroxylated iron oxide species.

The estimated surface load (%) was calculated based on the residual mass data by using pristine Fe_3_O_4_ as the reference and assigning the additional mass loss to surface-associated species formed during SBF immersion. Equation (2) was used to calculate the estimated surface load (EL%).

(2) $$EL\left(\%\right)=100-\frac{100\times {RM}_{SBF}}{{RM}_{Mag}}$$

where RM_SBF_ is the residual mass of a specific magnetite–SBF sample, and RM_Mag_ is the residual mass of pristine Fe_3_O_4_, namely 97.12%.

### 3.7. In Vitro Bioactivity Evaluation

**Figure 8:**
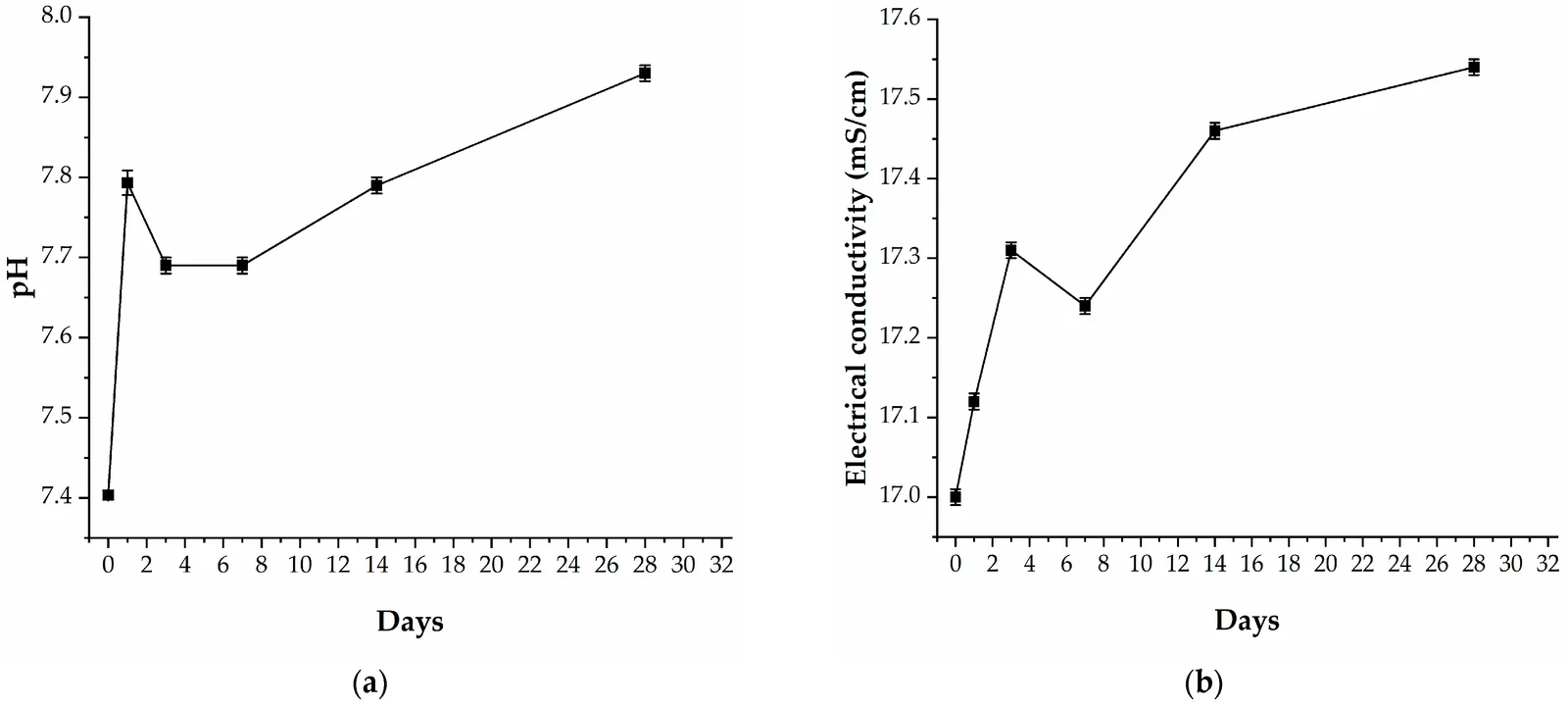
pH (**a**) and electrical conductivity (**b**) variation of SBF solution as a function of immersion time.

The pH and electrical conductivity variations of the SBF solution during magnetite immersion are presented in Figure 8. The initial pH of the SBF solution was 7.40. During the first 24 h of immersion, the pH increased to 7.79. After 3 days, a slight decrease was observed, and the pH remained close to 7.69 between days 3 and 7. At longer immersion times, the pH gradually increased, reaching 7.93 after 28 days. This trend shows that the contact between magnetite and SBF modifies the solution environment. The pH measurements alone cannot separate the contribution of ion exchange, surface hydroxylation, Ca–P deposition or other solution-mediated processes.

The initial increase in pH should therefore be interpreted only as an indication of an interfacial response between magnetite and SBF. Surface hydroxylation and ion-exchange reactions may contribute to this behavior. The slight decrease followed by the nearly constant pH between days 3 and 7 may reflect changes in the balance between ion release and ion consumption in solution, but it cannot be assigned to a specific mechanism without support from complementary analyses.

Regarding electrical conductivity, a slight increase was observed in the first days of immersion, followed by a small decrease around day 7, and then a gradual rise until day 28. These changes show that the ionic composition of the SBF solution varied during immersion. Conductivity measurements alone do not directly demonstrate Ca–P nucleation, apatite formation or ion incorporation into a surface layer. The pH and conductivity data were interpreted as indirect indicators of solution changes during SBF immersion and were correlated with the ED-XRF, FTIR-ATR and SEM-EDS results, which provide supporting evidence of Ca–P-containing surface deposits. Taken together with the ED-XRF, FTIR-ATR and SEM-EDS results, the in vitro bioactivity evaluation indicates that magnetite exhibits surface reactivity in SBF, associated with the formation of Ca–P-containing surface deposits. The pH and conductivity measurements were considered only as complementary evidence of changes occurring at the particle–solution interface and not as direct proof of bioactivity or apatite formation.

### 3.8. Cell Viability

**Figure 9:**
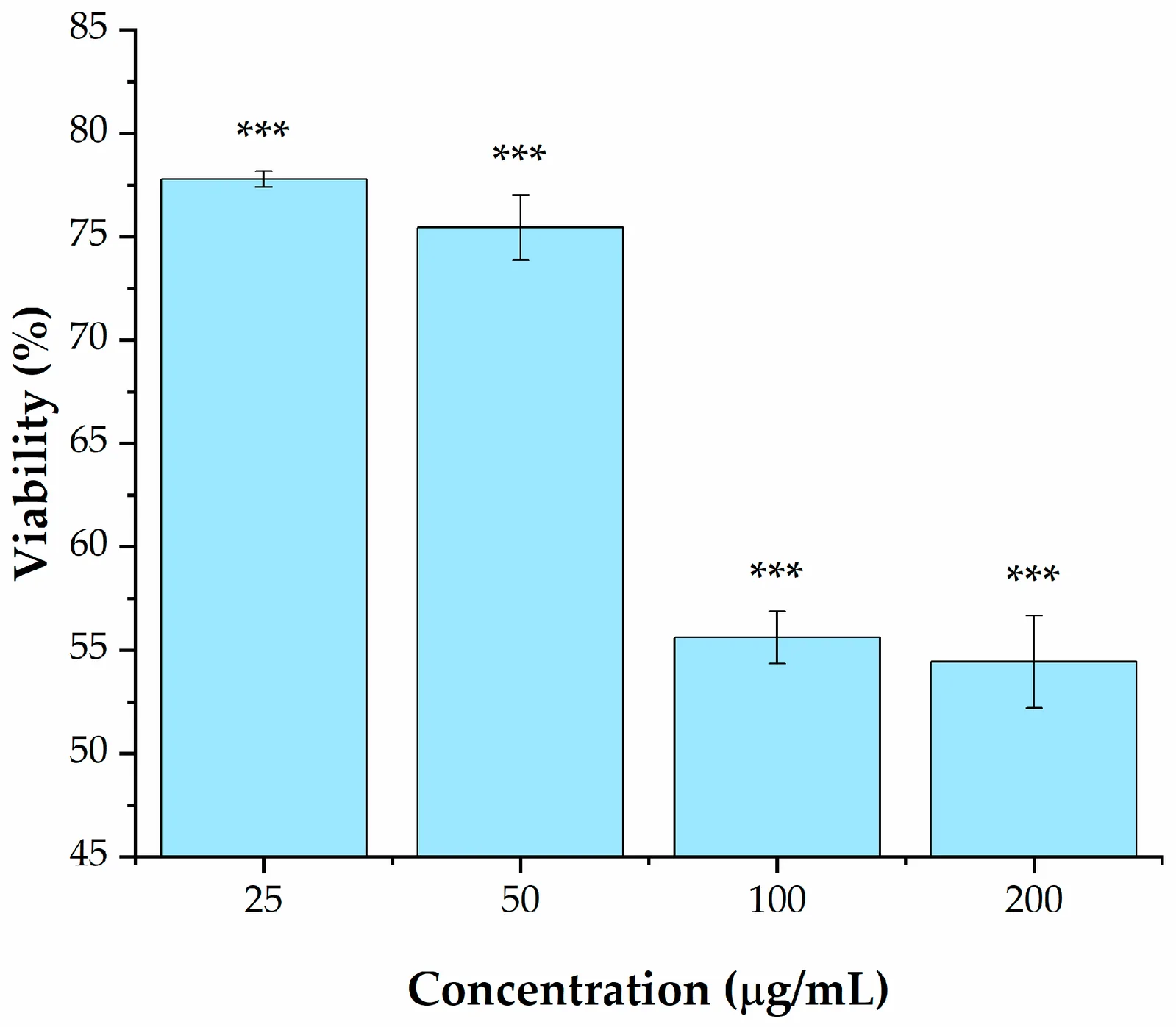
Cell viability of Fe_3_O_4__14SBF, corresponding to magnetite nanoparticles immersed in SBF for 14 days at concentrations of 25–200 µg/mL after 5 days of incubation with L929 fibroblast cells. Data are presented as means ± SD (*n* = 3). Statistical significance was determined using Student’s *t*-test (*** *p* < 0.001) compared to the negative control.

The Fe_3_O_4__14SBF sample was selected for cytocompatibility assessment as a representative intermediate stage of SBF-induced surface modification. At this immersion time, Ca–P-containing surface deposits were already observed, while the surface coverage was not as advanced as that observed after 28 days. The 28-day sample was not selected because it represented the most prolonged immersion condition and the most extensively modified surface, whereas the aim of the assay was to evaluate the cytocompatibility of a representative SBF-modified magnetite powder rather than to compare all immersion times. For this purpose, the tetrazolium salt-based MTT assay was used, which measures the total metabolic activity of the cells [91]. As shown in Figure 9, fibroblast viability was higher at lower Fe_3_O_4__14SBF concentrations and decreased when the concentration was increased.

For nanoparticle concentrations of 25 and 50 µg/mL, the viability was above the biocompatibility threshold of 70% described by ISO 10993-5 (Biological evaluation of medical devices—Part 5: Tests for in vitro cytotoxicity) [92]. At 100 and 200 µg/mL, cell viability decreased to about 55% of the negative control (*p* < 0.001). This decrease indicates that the higher Fe_3_O_4__14SBF concentrations reduced the metabolic activity of L929 fibroblast cells. This effect may be related to the higher number of particles in contact with the cells and to possible particle aggregation at higher concentrations.

Previous studies on iron oxide nanoparticles have shown that nanoparticle–cell interactions can be influenced by coating, aggregation state, colloidal stability in the culture medium and sedimentation near the cell surface [93,94,95].

Fe_3_O_4__14SBF showed acceptable cytocompatibility at 25 and 50 µg/mL, while higher concentrations reduced cell viability below the commonly accepted cytocompatibility threshold.

## 4. Conclusions

In this work, magnetite was synthesized by the chemical co-precipitation method, and its surface reactivity after immersion in SBF was evaluated.

XRD analysis supported the formation of magnetite as the main crystalline phase and showed that no major bulk crystalline changes occurred during SBF immersion. Considering the overlap between magnetite and maghemite diffraction peaks and the limitations of the employed techniques, minor surface-oxidized or hydroxylated iron oxide species cannot be completely excluded.

ED-XRF and SEM-EDS analyses supported a time-dependent surface modification during SBF immersion, characterized by an initial P-rich response followed by progressive Ca enrichment and the formation of Ca–P-containing deposits on the magnetite particle surfaces.

FTIR-ATR showed phosphate-related vibrations in the samples immersed in SBF, while the Fe-O bands maintained their characteristic positions. The formation of Ca–P-containing deposits was therefore interpreted based on the combined ED-XRF, SEM-EDS and FTIR-ATR results, rather than as direct evidence of a well-crystallized apatite layer.

TG-DSC measurements provided complementary information on adsorbed water, surface hydroxyl groups and thermal events associated with iron oxide behavior.

The results of DLS reveal moderately aggregated particles with a positive zeta potential, consistent with a hydrated surface that may influence interactions with ions from the SBF medium.

The pH and electrical conductivity variations indicated changes in the solution environment during immersion and were considered complementary indicators of interfacial processes, rather than direct evidence of bioactivity or apatite formation.

Cell viability tests indicated that Fe_3_O_4__14SBF concentrations of 25–50 µg/mL were well tolerated by L929 fibroblast cells, whereas higher concentrations of 100–200 µg/mL reduced cell viability.

The results indicate that the synthesized Fe_3_O_4_-based powder exhibits surface reactivity in SBF, associated with the formation of Ca–P-containing surface deposits, while maintaining its main crystalline structure. These findings suggest that co-precipitated magnetite may represent a useful component for the development of Fe_3_O_4_-based biomaterials for tissue engineering-related applications, provided that concentration-dependent cytocompatibility is considered.

## Figures and Tables

**Table 1:** Lattice parameters, average crystallite size, degree of crystallinity and agreement indices obtained by Rietveld refinement of the Fe_3_O_4_-indexed spinel iron oxide phase. Crystallite-size and lattice-parameter values are presented as means ± standard deviation.

Sample	Unit Cell Parameters		Average Crystallite Size [nm]	Degree of Crystallinity [%]	Agreement Indices		
	a = b = c [Å]	α = β = γ [°]			R_p_	R_wp_	χ^2^
Pristine Fe_3_O_4_	8.347 ± 0.001	90	9.94 ± 1.83	57.98	2.49	3.25	1.04
Fe_3_O_4__1SBF	8.354 ± 0.002	90	9.82 ± 2.07	48.84	2.79	3.93	1.05
Fe_3_O_4__3SBF	8.354 ± 0.003	90	6.40 ± 1.00	46.82	3.01	4.22	1.06
Fe_3_O_4__7SBF	8.349 ± 0.002	90	9.24 ± 1.81	49.41	2.09	3.14	1.04
Fe_3_O_4__14SBF	8.344 ± 0.002	90	9.04 ± 1.74	57.82	1.89	2.91	1.03
Fe_3_O_4__28SBF	8.356 ± 0.001	90	11.18 ± 3.01	57.96	2.32	3.45	1.05

**Table 2:** Selected ED-XRF elemental contents (wt.%) of pristine Fe_3_O_4_ and Fe_3_O_4_ samples after 1, 14, and 28 days of SBF immersion.

Sample	Analyte	Result (wt.%)	Standard Deviation	Line	Intensity (cps/μA)
Pristine Fe_3_O_4_	Fe	99.527	0.121	Fe Kα	8810.550
Fe_3_O_4__1SBF	Fe	98.095	0.128	Fe Kα	7610.043
	P	1.073	0.245	P Kα	0.776
	Ca	0.433	0.015	Ca Kα	9.108
Fe_3_O_4__14SBF	Fe	97.291	0.120	Fe Kα	8469.080
	P	1.457	0.246	P Kα	1.197
	Ca	0.649	0.018	Ca Kα	15.292
Fe_3_O_4__28SBF	Fe	96.210	0.119	Fe Kα	8396.544
	P	2.209	0.274	P Kα	1.831
	Ca	1.152	0.025	Ca Kα	26.997

Note: Only Fe, P, and Ca are reported because they are directly relevant to the interpretation of SBF-induced compositional changes. The original semi-quantitative values were retained without renormalization. The unreported fraction corresponds to minor or trace signals excluded from the interpretation.

**Table 3:** Results of DLS analysis of the Fe_3_O_4_ sample.

Measurement	Zeta Potential (mV)	Hydrodynamic Diameter (nm)	Polydispersity Index (PDI)
1	22.70	211.10	0.23
2	21.22	214.20	0.19
3	15.92	222.80	0.24
Mean ± SD	19.95 ± 3.56	216.03 ± 6.06	0.22 ± 0.02

**Table 4:** The principal numeric data from the thermal analysis of magnetite samples.

Sample	ML_1_ (%)	Endo T_1_ (°C)	ML_2_ (%)	Exo T_2_ (°C)	ML_3_ (%)	Exo T_3_ (°C)	RM (%)	EL (%)
Pristine Fe_3_O_4_	0.85	75.9	1.34	331.8	0.69	522.0	97.12	-
Fe_3_O_4__1SBF	1.05	74.0	2.21	269.1 338.1	1.18	644.9	95.55	1.62
Fe_3_O_4__3SBF	1.14	79.9	1.95	277.2 340.0	1.95	662.1	96.38	0.76
Fe_3_O_4__7SBF	1.31	62.1	3.71	285.0 411.0	1.39	664.2	93.64	3.58
Fe_3_O_4__14SBF	1.21	77.4	2.01	271.8 328.0	0.98	655.0	95.82	1.34
Fe_3_O_4__28SBF	1.28	71.0	1.50	-	0.57	669.1	96.64	0.49

Note: ML_1_, ML_2_, and ML_3_ correspond to the mass losses recorded in the RT–180 °C, 180–480 °C, and 480–900 °C temperature ranges, respectively. Endo T_1_, Exo T_2_, and Exo T_3_ denote the temperatures of the associated thermal events, with Exo T_3_ being associated with iron oxide oxidation/phase transformation processes during heating in air. RM indicates the residual mass and EL the estimated load.

## Data Availability

The original contributions presented in the study are included in the article, further inquiries can be directed to the corresponding author.

## Supplementary Materials

The following supporting information can be downloaded at https://www.mdpi.com/article/10.3390/jfb17080393/s1, Figure S1: ED-XRF spectra of the Fe_3_O_4_ powders: before (a) and after immersion in SBF for 1 day (b), 14 days (c) and 28 days (d); Figure S2: Zoom-in detail of the TG-DSC curves showing the low-temperature endothermic events associated with dehydration of the samples.

## References

[1] Baig N., Kammakakam I., Falath W. (2021). Nanomaterials: A Review of Synthesis Methods, Properties, Recent Progress, and Challenges. Mater. Adv..

[2] Haleem A., Javaid M., Singh R.P., Rab S., Suman R. (2023). Applications of Nanotechnology in Medical Field: A Brief Review. Glob. Health J..

[3] Kazi R.N.A., Hasani I.W., Khafaga D.S.R., Kabba S., Farhan M., Aatif M., Muteeb G., Fahim Y.A. (2025). Nanomedicine: The Effective Role of Nanomaterials in Healthcare from Diagnosis to Therapy. Pharmaceutics.

[4] Mohammed H.B., Rayyif S.M.I., Curutiu C., Birca A.C., Oprea O.-C., Grumezescu A.M., Ditu L.-M., Gheorghe I., Chifiriuc M.C., Mihaescu G. (2021). Eugenol-Functionalized Magnetite Nanoparticles Modulate Virulence and Persistence in *Pseudomonas aeruginosa* Clinical Strains. Molecules.

[5] Sengupta J. (2020). Application of Carbon Nanomaterials in the Electronic Industry. Handbook of Nanomaterials for Manufacturing Applications.

[6] Słoma M. (2023). 3D Printed Electronics with Nanomaterials. Nanoscale.

[7] Payal, Pandey P. (2022). Role of Nanotechnology in Electronics: A Review of Recent Developmentsand Patents. Recent Pat. Nanotechnol..

[8] Nishu, Kumar S. (2023). Smart and Innovative Nanotechnology Applications for Water Purification. Hybrid. Adv..

[9] Zango Z.U., Garba A., Shittu F.B., Imam S.S., Haruna A., Zango M.U., Wadi I.A., Bello U., Adamu H., Keshta B.E. (2025). A State-of-the-Art Review on Green Synthesis and Modifications of ZnO Nanoparticles for Organic Pollutants Decomposition and CO_2_ Conversion. J. Hazard. Mater. Adv..

[10] Han S., Kim J., Ko S.H. (2021). Advances in Air Filtration Technologies: Structure-Based and Interaction-Based Approaches. Mater. Today Adv..

[11] Mohammed H., Mia M.F., Wiggins J., Desai S. (2025). Nanomaterials for Energy Storage Systems—A Review. Molecules.

[12] Lotti Diaz L.M., Hughes K., Ganesan M., Tenchov R., Iyer K.A., Ralhan K., Bird R.E., Baranwal B.S., Ivanov J.M., Zhou Q.A. (2025). Nanotechnology in Action: A Broad Perspective on Nanoscale Materials for Energy Applications. ACS Appl. Energy Mater..

[13] Gohar O., Zubair Khan M., Bibi I., Bashir N., Tariq U., Bakhtiar M., Ramzan Abdul Karim M., Ali F., Bilal Hanif M., Motola M. (2024). Nanomaterials for Advanced Energy Applications: Recent Advancements and Future Trends. Mater. Des..

[14] Pozzi M., Jonak Dutta S., Kuntze M., Bading J., Rüßbült J.S., Fabig C., Langfeldt M., Schulz F., Horcajada P., Parak W.J. (2024). Visualization of the High Surface-to-Volume Ratio of Nanomaterials and Its Consequences. J. Chem. Educ..

[15] Nguyen M.D., Tran H.-V., Xu S., Lee T.R. (2021). Fe_3_O_4_ Nanoparticles: Structures, Synthesis, Magnetic Properties, Surface Functionalization, and Emerging Applications. Appl. Sci..

[16] Kristanti R.A., Hadibarata T., Niculescu A.-G., Mihaiescu D.E., Grumezescu A.M. (2025). Nanomaterials for Persistent Organic Pollutants Decontamination in Water: Mechanisms, Challenges, and Future Perspectives. Nanomaterials.

[17] Sohail A. (2025). Progress in Nanomaterials: Synthesis, Characterization, and Applications. Next Nanotechnol..

[18] Laurent S., Forge D., Port M., Roch A., Robic C., Vander Elst L., Muller R.N. (2010). Magnetic Iron Oxide Nanoparticles: Synthesis, Stabilization, Vectorization, Physicochemical Characterizations, and Biological Applications. Chem. Rev..

[19] Pankhurst Q.A., Connolly J., Jones S.K., Dobson J. (2003). Applications of Magnetic Nanoparticles in Biomedicine. J. Phys. D Appl. Phys..

[20] Baldea I., Iacoviță C., Gurgu R.A., Vizitiu A.S., Râzniceanu V., Mitrea D.R. (2025). Magnetic Hyperthermia with Iron Oxide Nanoparticles: From Toxicity Challenges to Cancer Applications. Nanomaterials.

[21] Aboushoushah S.F.O. (2025). Iron Oxide Nanoparticles Enhancing Magnetic Resonance Imaging: A Review of the Latest Advancements. J. Sci. Adv. Mater. Devices.

[22] Ghazi R., Ibrahim T.K., Nasir J.A., Gai S., Ali G., Boukhris I., Rehman Z. (2025). Iron Oxide Based Magnetic Nanoparticles for Hyperthermia, MRI and Drug Delivery Applications: A Review. RSC Adv..

[23] Salehirozveh M., Dehghani P., Mijakovic I. (2024). Synthesis, Functionalization, and Biomedical Applications of Iron Oxide Nanoparticles (IONPs). J. Funct. Biomater..

[24] Mdlovu N.V., Lin K.-S., Weng M.-T., Lin Y.-S., Liu S.-Y. (2022). Preparation and In-Vitro/in-Vivo Evaluation of Doxorubicin-Loaded Magnetic SBA-15 Nanocomposites from Rice Husk for Enhancing Therapeutic Efficacy. Colloids Surf. B Biointerfaces.

[25] Motelica L., Vasile B.S., Ficai D., Oprea O.-C., Surdu V.-A., Trusca R.D., Spoiala A., Voicu G., Anghelache M., Marta D.S. (2026). Smart Magnetic Drug Delivery System for Targeted, Intracellular Delivery of Irinotecan and Platinum-Based Antitumoral Drugs. Materials.

[26] Angela S., Ludmila M., Cornelia-Ioana I., Denisa F., Cristina C., Natalia P., Sabina G., Mateusz M., Joanna K., Adrian-Vasile S. (2024). Aminoacid Functionalised Magnetite Nanoparticles Fe_3_O_4_@AA (AA = Ser, Cys, Pro, Trp) as Biocompatible Magnetite Nanoparticles with Potential Therapeutic Applications. Sci. Rep..

[27] Hadadian Y., Masoomi H., Dinari A., Ryu C., Hwang S., Kim S., Cho B.K., Lee J.Y., Yoon J. (2022). From Low to High Saturation Magnetization in Magnetite Nanoparticles: The Crucial Role of the Molar Ratios Between the Chemicals. ACS Omega.

[28] Meng Y.Q., Shi Y.N., Zhu Y.P., Liu Y.Q., Gu L.W., Liu D.D., Ma A., Xia F., Guo Q.Y., Xu C.C. (2024). Recent Trends in Preparation and Biomedical Applications of Iron Oxide Nanoparticles. J. Nanobiotechnol..

[29] Motelica L., Voicu G., Chircov C., Surdu A.V., Trusca R.D., Vasile B.S., Ficai D., Oprea O.C., Marta D.S., Peteu V.-E. (2025). Aspartic Acid Functionalized Magnetic Nanoparticles for Enhanced Internalization in Tumoral Cell. J. Aust. Ceram. Soc..

[30] Ganapathe L.S., Mohamed M.A., Mohamad Yunus R., Berhanuddin D.D. (2020). Magnetite (Fe_3_O_4_) Nanoparticles in Biomedical Application: From Synthesis to Surface Functionalisation. Magnetochemistry.

[31] Gupta A.K., Gupta M. (2005). Synthesis and Surface Engineering of Iron Oxide Nanoparticles for Biomedical Applications. Biomaterials.

[32] Gherasim O., Popescu R.C., Grumezescu V., Mogoșanu G.D., Mogoantă L., Iordache F., Holban A.M., Vasile B.Ș., Bîrcă A.C., Oprea O.-C. (2021). MAPLE Coatings Embedded with Essential Oil-Conjugated Magnetite for Anti-Biofilm Applications. Materials.

[33] Dudchenko N., Pawar S., Perelshtein I., Fixler D. (2022). Magnetite Nanoparticles: Synthesis and Applications in Optics and Nanophotonics. Materials.

[34] Atrei A., Mahdizadeh F.F., Baratto M.C., Scala A. (2021). Effect of Citrate on the Size and the Magnetic Properties of Primary Fe_3_O_4_ Nanoparticles and Their Aggregates. Appl. Sci..

[35] Buffle J., Leppard G.G. (1995). Characterization of Aquatic Colloids and Macromolecules. 1. Structure and Behavior of Colloidal Material. Environ. Sci. Technol..

[36] Cornell R.M., Schwertmann U. (2003). The Iron Oxides: Structure, Properties, Reactions, Occurences and Uses.

[37] Baino F., Yamaguchi S. (2020). The Use of Simulated Body Fluid (SBF) for Assessing Materials Bioactivity in the Context of Tissue Engineering: Review and Challenges. Biomimetics.

[38] Dong W., Matsukawa Y., Long Y., Hayashi Y., Nakamura J., Suzuki K., Ohtsuki C. (2024). Revised Method for Preparation of Simulated Body Fluid for Assessment of the Apatite-Forming Ability of Bioactive Materials: Proposal of Mixing Two Stock Solutions. RSC Adv..

[39] Kokubo T., Takadama H. (2006). How Useful Is SBF in Predicting in Vivo Bone Bioactivity?. Biomaterials.

[40] Szewczyk A., Skwira-Rucińska A., Osińska M., Prokopowicz M. (2024). The Apatite-Forming Ability of Bioactive Glasses—A Comparative Study in Human Serum and Kokubo’s Simulated Body Fluid. Ceram. Int..

[41] Nguyen A.K., Nelson S.B., Skoog S.A., Jaipan P., Petrochenko P.E., Kaiser A., Lo L., Moreno J., Narayan R.J., Goering P.L. (2023). Effect of Simulated Body Fluid Formulation on Orthopedic Device Apatite-forming Ability Assessment. J. Biomed. Mater. Res..

[42] Valverde T.M., Dos Santos V.M.R., Viana P.I.M., Costa G.M.J., De Goes A.M., Sousa L.R.D., Xavier V.F., Vieira P.M.D.A., De Lima Silva D., Domingues R.Z. (2024). Novel Fe_3_O_4_ Nanoparticles with Bioactive Glass–Naproxen Coating: Synthesis, Characterization, and In Vitro Evaluation of Bioactivity. Int. J. Mol. Sci..

[43] Liu H., Siani P., Bianchetti E., Zhao J., Di Valentin C. (2021). Multiscale Simulations of the Hydration Shells Surrounding Spherical Fe_3_ O_4_ Nanoparticles and Effect on Magnetic Properties. Nanoscale.

[44] Orozco-Henao J.M., Alí F.L., Azcárate J.C., Robledo Candia L.D., Pasquevich G., Mendoza Zélis P., Haas B., Coogan K., Kirmse H., Koch C.T. (2024). Oxidation Kinetics of Magnetite Nanoparticles: Blocking Effect of Surface Ligands and Implications for the Design of Magnetic Nanoheaters. Chem. Mater..

[45] Turrina C., Klassen A., Milani D., Rojas-González D.M., Ledinski G., Auer D., Sartori B., Cvirn G., Mela P., Berensmeier S. (2023). Superparamagnetic Iron Oxide Nanoparticles for Their Application in the Human Body: Influence of the Surface. Heliyon.

[46] Dridi A., Riahi K.Z., Somrani S. (2021). Mechanism of Apatite Formation on a Poorly Crystallized Calcium Phosphate in a Simulated Body Fluid (SBF) at 37 °C. J. Phys. Chem. Solids.

[47] Islam M.T., Felfel R.M., Abou Neel E.A., Grant D.M., Ahmed I., Hossain K.M.Z. (2017). Bioactive Calcium Phosphate–Based Glasses and Ceramics and Their Biomedical Applications: A Review. J. Tissue Eng..

[48] Li Y., Liu C., Liu W., Cheng X., Zhang A., Zhang S., Liu C., Li N., Jian X. (2021). Apatite Formation Induced by Chitosan/Gelatin Hydrogel Coating Anchored on Poly(Aryl Ether Nitrile Ketone) Substrates to Promote Osteoblastic Differentiation. Macromol. Biosci..

[49] He Z., Jiao C., Wu J., Gu J., Liang H., Shen L., Yang Y., Tian Z., Wang C., Jiang Q. (2023). Zn-Doped Chitosan/Alginate Multilayer Coatings on Porous Hydroxyapatite Scaffold with Osteogenic and Antibacterial Properties. Int. J. Bioprinting.

[50] Hench L.L., Polak J.M. (2002). Third-Generation Biomedical Materials. Science.

[51] Popescu R.C., Andronescu E., Vasile B.S. (2019). Recent Advances in Magnetite Nanoparticle Functionalization for Nanomedicine. Nanomaterials.

[52] Nguyen M.D., Hoijang S., Yarinia R., Ariza Gonzalez M., Mandal S., Tran Q.M., Chinwangso P., Lee T.R. (2025). Magnetic Iron Oxide Nanoparticles: Advances in Synthesis, Mechanistic Understanding, and Magnetic Property Optimization for Improved Biomedical Performance. Nanomaterials.

[53] Shin K., Acri T., Geary S., Salem A.K. (2017). Biomimetic Mineralization of Biomaterials Using Simulated Body Fluids for Bone Tissue Engineering and Regenerative Medicine. Tissue Eng. Part A.

[54] Jafari Eskandari M., Hasanzadeh I. (2021). Size-Controlled Synthesis of Fe_3_O_4_ Magnetic Nanoparticles via an Alternating Magnetic Field and Ultrasonic-Assisted Chemical Co-Precipitation. Mater. Sci. Eng. B.

[55] Kokubo T., Kushitani H., Sakka S., Kitsugi T., Yamamuro T. (1990). Solutions Able to Reproduce in Vivo Surface-structure Changes in Bioactive Glass-ceramic A-W^3^. J. Biomed. Mater. Res..

[56] Ismail A.M., Tiama T.M., Farghaly A., Elhaes H., Ibrahim M.A. (2023). Assessment of the Functionalization of Chitosan/Iron Oxide Nanoparticles. Biointerface Res. Appl. Chem..

[57] Matli P.R., Zhou X., Shiyu D., Huang Q. (2015). Fabrication, Characterization, and Magnetic Behavior of Porous ZnFe_2_O_4_ Hollow Microspheres. Int. Nano Lett..

[58] Velásquez A.A., Urquijo J.P. (2021). Synthesis and Characterization of Magnetite-Maghemite Nanoparticles in Presence of Polyethylene Glycol Obtained by Mechanical Milling. Mater. Sci. Eng. B.

[59] Chircov C., Dumitru I.A., Vasile B.S., Oprea O.-C., Holban A.M., Popescu R.C. (2023). Microfluidic Synthesis of Magnetite Nanoparticles for the Controlled Release of Antibiotics. Pharmaceutics.

[60] Zanotto C., Ratuchne F., Guimarães De Castro E., Teixeira Marques P. (2019). Structural Characterization of Magnetite and Its Influence on the Formation of Composites with Polyaniline. Orbital Electron. J. Chem..

[61] Roacho-Pérez J.A., Ruiz-Hernandez F.G., Chapa-Gonzalez C., Martínez-Rodríguez H.G., Flores-Urquizo I.A., Pedroza-Montoya F.E., Garza-Treviño E.N., Bautista-Villareal M., García-Casillas P.E., Sánchez-Domínguez C.N. (2020). Magnetite Nanoparticles Coated with PEG 3350-Tween 80: In Vitro Characterization Using Primary Cell Cultures. Polymers.

[62] Kristl M., Ostroško U., Ban I., Petrinić I., Stergar J. (2024). Thermal Study of APTES-Functionalized Magnetite Nanoparticles with Citric Acid and Polyacrylic Acid for Advanced Forward Osmosis Systems. J. Therm. Anal. Calorim..

[63] Hormozinezhad Z., Gorjizadeh M., Taheri N., Sayyahi S. (2018). [Fe_3_O_4_@SiO_2_@(C_3_H_6_)2-(Imidazolium)2-Butyl][MnCl_4_^2−^] as a Novel Nanomagnetic Catalyst for the One-Pot Synthesis of Tetrahydrobenzo[b]Pyran Derivatives. Bull. Chem. Soc. Ethiop..

[64] Răcuciu M., Oancea S. (2019). ATR-FTIR versus Raman Spectroscopy Used for Structural Analyses of the Iron Oxide Nanoparticles. Rom. Rep. Phys..

[65] Schott J.A., Do-Thanh C.-L., Shan W., Puskar N.G., Dai S., Mahurin S.M. (2021). FTIR Investigation of the Interfacial Properties and Mechanisms of CO_2_ Sorption in Porous Ionic Liquids. Green Chem. Eng..

[66] Tkachenko Y., Niedzielski P. (2022). FTIR as a Method for Qualitative Assessment of Solid Samples in Geochemical Research: A Review. Molecules.

[67] Ahmad S., Ni H., Al-Mubaddel F.S., Rizk M.A., Ammar M.B., Khan A.U., Almarhoon Z.M., Alanazi A.A., Zaki M.E.A. (2025). Magnetic Properties of Different Phases Iron Oxide Nanoparticles Prepared by Micro Emulsion-Hydrothermal Method. Sci. Rep..

[68] Pasieczna-Patkowska S., Cichy M., Flieger J. (2025). Application of Fourier Transform Infrared (FTIR) Spectroscopy in Characterization of Green Synthesized Nanoparticles. Molecules.

[69] Gomes E.D.S., Lima A.M.D.O., Gavinho S.R., Graça M.P.F., Devesa S., Macêdo A.A.M. (2023). Influence of Polymeric Blends on Bioceramics of Hydroxyapatite. Crystals.

[70] Guzman-Rocha D.A., Cano-Gonzalez M.E., Garcia-Contreras R. (2025). Physicochemical Properties of Magnetic Scaffolds with Hydroxyapatite Nanoparticles and Chitosan. MRS Adv..

[71] Lee H., Lee C.Y., Hwang J.K., Chung W.J. (2017). P_2_O_5_-ZnO-SiO_2_-R_2_O Glass Frit Materials for Hermetic Sealing of Dye-Sensitized Solar Cells. J. Korean Ceram. Soc..

[72] Salamat S., Younesi H., Bahramifar N. (2017). Synthesis of Magnetic Core–Shell Fe_3_ O_4_ @TiO_2_ Nanoparticles from Electric Arc Furnace Dust for Photocatalytic Degradation of Steel Mill Wastewater. RSC Adv..

[73] Charoensuk T., Sirisathitkul C., Tangwatanakul W., Pinitsoontorn S., Boonyang U. (2016). Magnetic Phase Transitions in Macro/Mesoporous Bioactive Glass by Ferric Nitrate Addition in Sol-Gel Synthesis. J. Ceram. Sci. Tech..

[74] Vats V., Melton G., Islam M., Krishnan V.V. (2023). FTIR Spectroscopy as a Convenient Tool for Detection and Identification of Airborne Cr(VI) Compounds Arising from Arc Welding Fumes. J. Hazard. Mater..

[75] Chircov C., Bejenaru I.T., Nicoară A.I., Bîrcă A.C., Oprea O.C., Tihăuan B. (2022). Chitosan-Dextran-Glycerol Hydrogels Loaded with Iron Oxide Nanoparticles for Wound Dressing Applications. Pharmaceutics.

[76] Smiri M., Guey F., Chemingui H., Dekhil A.B., Elarbaoui S., Hafiane A. (2020). Remove of Humic Acid From Water Using Magnetite Nanoparticles. Eur. J. Adv. Chem. Res..

[77] Govindan R., Karthi S., Kumar G.S., Girija E.K. (2020). Development of Fe_3_ O_4_ Integrated Polymer/Phosphate Glass Composite Scaffolds for Bone Tissue Engineering. Mater. Adv..

[78] Tombacz E., Majzik A., Horvat Z.S., Illes E. (2006). Magnetite in Aqueous Medium: Coating Its Surface and Surface Coated with It. Romanian Rep. Phys..

[79] Sun Z.-X., Su F.-W., Forsling W., Samskog P.-O. (1998). Surface Characteristics of Magnetite in Aqueous Suspension. J. Colloid Interface Sci..

[80] Lim J., Yeap S.P., Che H.X., Low S.C. (2013). Characterization of Magnetic Nanoparticle by Dynamic Light Scattering. Nanoscale Res. Lett..

[81] Alimohammadi V., Seyyed Ebrahimi S.A., Kashanian F., Lalegani Z., Habibi-Rezaei M., Hamawandi B. (2022). Hydrophobic Magnetite Nanoparticles for Bioseparation: Green Synthesis, Functionalization, and Characterization. Magnetochemistry.

[82] Puiu R.A., Balaure P.C., Constantinescu E., Grumezescu A.M., Andronescu E., Oprea O.-C., Vasile B.S., Grumezescu V., Negut I., Nica I.C. (2021). Anti-Cancer Nanopowders and MAPLE-Fabricated Thin Films Based on SPIONs Surface Modified with Paclitaxel Loaded β-Cyclodextrin. Pharmaceutics.

[83] Kalska-Szostko B., Wykowska U., Satula D., Nordblad P. (2015). Thermal Treatment of Magnetite Nanoparticles. Beilstein J. Nanotechnol..

[84] Ficai D., Ficai A., Vasile B.S., Ficai M., Oprea O., Guran C., Andronescu E. (2011). Synthesis of Rod-like Magnetite by Using Low Magnetic Field. Dig. J. Nanomater. Biostructures.

[85] Li Z., Chanéac C., Berger G., Delaunay S., Graff A., Lefèvre G. (2019). Mechanism and Kinetics of Magnetite Oxidation under Hydrothermal Conditions. RSC Adv..

[86] Schimanke G. (2000). In Situ XRD Study of the Phase Transition of Nanocrystalline Maghemite (γ-Fe_2_O_3_) to Hematite (α-Fe_2_O_3_). Solid State Ion..

[87] Chircov C., Matei M.-F., Neacșu I.A., Vasile B.S., Oprea O.-C., Croitoru A.-M., Trușcă R.-D., Andronescu E., Sorescu I., Bărbuceanu F. (2021). Iron Oxide–Silica Core–Shell Nanoparticles Functionalized with Essential Oils for Antimicrobial Therapies. Antibiotics.

[88] Ament K., Wagner D.R., Meij F.E., Wagner F.E., Breu J. (2020). High Temperature Stable Maghemite Nanoparticles Sandwiched between Hectorite Nanosheets. Z. Anorg. Allge Chem..

[89] Stoia M., Istratie R., Păcurariu C. (2016). Investigation of Magnetite Nanoparticles Stability in Air by Thermal Analysis and FTIR Spectroscopy. J. Therm. Anal. Calorim..

[90] Dorcioman G., Hudiță A., Gălățeanu B., Craciun D., Mercioniu I., Oprea O.C., Neguț I., Grumezescu V., Grumezescu A.M., Dițu L.M. (2022). Magnetite-Based Nanostructured Coatings Functionalized with Nigella Sativa and Dicloxacillin for Improved Wound Dressings. Antibiotics.

[91] Ghasemi M., Turnbull T., Sebastian S., Kempson I. (2021). The MTT Assay: Utility, Limitations, Pitfalls, and Interpretation in Bulk and Single-Cell Analysis. Int. J. Mol. Sci..

[92] (2009). Biological Evaluation of Medical Devices—Part 5: Tests for In Vitro Cytotoxicity.

[93] Safi M., Courtois J., Seigneuret M., Conjeaud H., Berret J.-F. (2011). The Effects of Aggregation and Protein Corona on the Cellular Internalization of Iron Oxide Nanoparticles. Biomaterials.

[94] Safi M., Berret J.-F. (2010). The Role of the Coating and Aggregation State in the Interactions between Iron Oxide Nanoparticles and 3T3 Fibroblasts. Phys. Procedia.

[95] Hinderliter P.M., Minard K.R., Orr G., Chrisler W.B., Thrall B.D., Pounds J.G., Teeguarden J.G. (2010). ISDD: A Computational Model of Particle Sedimentation, Diffusion and Target Cell Dosimetry for in Vitro Toxicity Studies. Part Fibre Toxicol..

