# Supplementary material for: Synthesis, Characterization and In Vitro Bioactivity of Magnetite Nanoparticles Obtained by Co-Precipitation
Source: J Funct Biomater. 2026 Aug 9;17(8):393. doi: 10.3390/jfb17080393 (PMC13514853; doi:10.3390/jfb17080393)
Supplement: Supplementary file 1 [file jfb-17-00393-s001.zip › jfb-4386277-supplementary.pdf]

Supplementary Materials

Article

Synthesis, Characterization and In Vitro Bioactivity of Magnetite Nanoparticles Obtained by Co-Precipitation

Marian Rascov [1,*], Angela Spoiala [2,3], Ludmila Motelica [2,3,4], Roxana-Doina Trusca [2], Cristina Chircov [1,2], Roxana Cristina Popescu [5], Otilia-Ruxandra Radacina [2], Vasile-Adrian Surdu [6], Denisa Ficai [2,4,7], Ovidiu-Cristian Oprea [2,4,7], Anton Ficai [1,2,3,4], Ecaterina Andronescu [1,2,4,8,*] and Claudiu Stefan Turculet [9,10]

1 Department of Science and Engineering of Oxide Materials and Nanomaterials, Faculty of Chemical Engineering and Biotechnologies, National University of Science and Technology Politehnica Bucharest, Gh. Polizu 1–7, 011061 Bucharest, Romania; marian.rascov@stud.chimie.upb.ro (M.R.); cristina.chircov@upb.ro (C.C.); anton.ficai@upb.ro (A.F.); ecaterina.andronescu@upb.ro (E.A.)

2 National Research Center for Micro and Nanomaterials, National University of Science and Technology Politehnica Bucharest, Splaiul Independentei 313, 060042 Bucharest, Romania; angela.spoiala@upb.ro (A.S.); ludmila.motelica@upb.ro (L.M.); roxana_doina.trusca@upb.ro (R.-D.T.); otilia.radacina@upb.ro (O.-R.R.); denisa.ficai@upb.ro (D.F.); ovidiu.oprea@upb.ro (O.-C.O.)

3 Research Center for Advanced Materials, Products and Processes, National University of Science and Technology Politehnica Bucharest, Splaiul Independentei 313, 060042 Bucharest, Romania

4 Academy of Romanian Scientists, Ilfov St. 3, 050044 Bucharest, Romania

5 Department of Life and Environmental Physics, National Institute for R&D in Physics and Nuclear Engineering "Horia Hulubei", Reactorului St., 30, 077125 Magurele, Romania

6 Department of Materials Science, Faculty of Materials Science and Engineering, Transilvania University of Brasov, 29 Eroilor Blvd., 500036 Brasov, Romania; vasile.surdu@unitbv.ro (V.-A.S.)

7 Department of Inorganic Chemistry, Physical Chemistry and Electrochemistry, Faculty of Chemical Engineering and Biotechnologies, National University of Science and Technology Politehnica Bucharest, Gh. Polizu 1–7, 011061 Bucharest, Romania

8 Technical Sciences Academy of Romania, 26 Dacia Blvd., 010414 Bucharest, Romania

9 Faculty of Medicine, "Carol Davila" University of Medicine and Pharmacy, Eroii Sanitari St. 8, 050474 Bucharest, Romania; claudiu.turculet@umfcd.ro (C.S.T.)

10 General Surgery Department, Clinical Emergency Hospital of Bucharest, 105402 Bucharest, Romania

* Correspondence: marian.rascov@stud.chimie.upb.ro (M.R.); ecaterina.andronescu@upb.ro (E.A.)

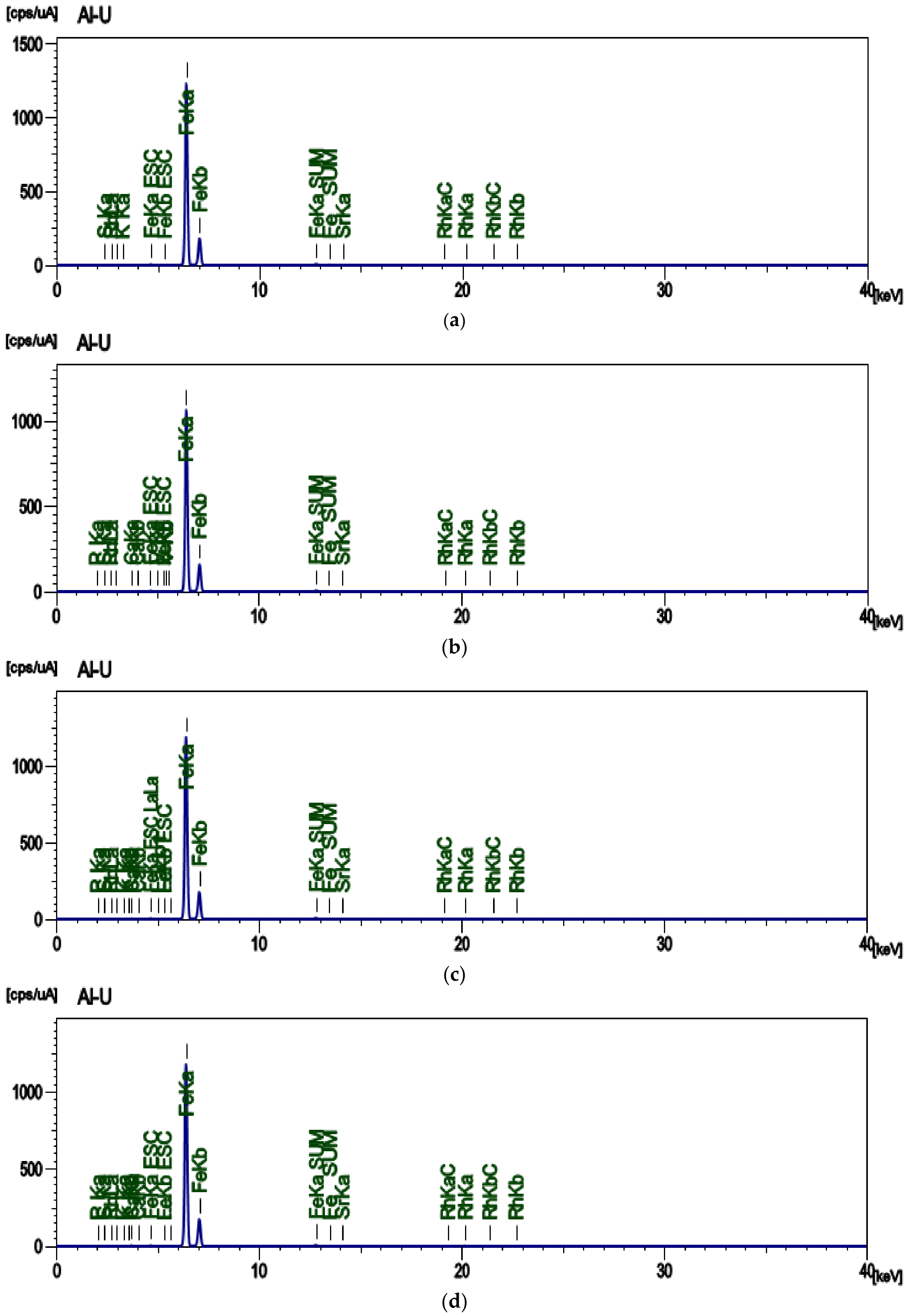

Figure S1. ED-XRF spectra of the Fe_3O_4 powders: before (a) and after immersion in SBF for 1 day (b), 14 days (c) and 28 days (d).

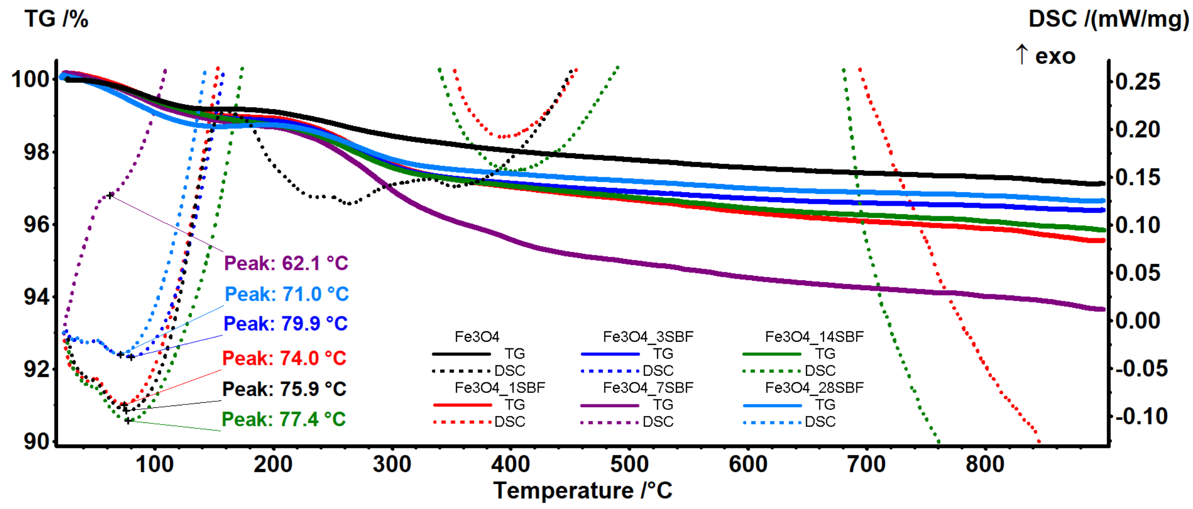

Figure S2. Zoom-in detail of the TG-DSC curves showing the low-temperature endothermic events associated with dehydration of the samples.
